# Radiolysis Derivatives from *p*-Coumaric Acid via Gamma Irradiation and Their Anti-Inflammatory Activities

**DOI:** 10.3390/molecules31101630

**Published:** 2026-05-12

**Authors:** Ah-Reum Han, Ha-Yeon Song, Gyeong Han Jeong, Euna Choi, Yu Jung Min, So-Yeon Kim, So-Yeun Woo, Chang Hyun Jin, Eui-Baek Byun, Hyoung-Woo Bai

**Affiliations:** 1 Advanced Radiation Technology Institute, Korea Atomic Energy Research Institute, Jeongeup-si 56212, Jeonbuk-do, Republic of Korea; 2 Department of Plant Resources, College of Industrial Sciences, Kongju National University, Yesan-gun 32439, Chungcheongnam-do, Republic of Korea; 3 Department of Food Science and Technology, College of Industrial Sciences, Kongju National University, Yesan-gun 32439, Chungcheongnam-do, Republic of Korea

**Keywords:** phenolic acid, solvent radiolysis, hydroxymethyl radicals, structure modification, cytokine regulation

## Abstract

Gamma irradiation serves as a robust platform for the structural diversification of natural compounds, utilizing high-energy reactions with free radicals to generate novel scaffolds with improved biological properties. In present study, *p*-coumaric acid was exposed to ionizing radiation at various doses to induce molecular transformations. Significant degradation of the precursor was confirmed at a dose of 60 kGy, which provided the optimal range for the generation of radiolysis products. The chromatographic fraction of the resulting mixture afforded two novel derivatives, **1** and **2**, along with a known analog **3**. Comprehensive spectroscopic characterization assigned their structures as (3*R*,4*R*)-3-hydroxymethyl-4-(4-hydroxyphenyl)-dihydrofuran-2(3*H*)one (**1**), (3R*,4S*)-3-hydroxymethyl-4-(4-hydroxyphenyl)-dihydrofuran-2(3*H*)-one (**2**), and (4*S*)-4-(4-hydroxyphenyl)-dihydrofuran-2(3*H*)-one (**3**). The anti-inflammatory effects of the isolates were evaluated using lipopolysaccharide-stimulated RAW264.7 macrophages. While neither the parent compound *p*-coumaric acid nor its derivatives exhibited significant cytotoxicity at concentrations up to 40 μM, their anti-inflammatory potencies varied significantly. Notably, compound **1** exhibited potent inhibitory effects on pro-inflammatory signaling, significantly inhibiting the production of TNF-α, IL-6, and IL-12p70, surpassing the bioactivity of the parent compound. Compound **2** displayed a similar, attenuated inhibitory trend, suppressing the secretion of TNF-α and IL-12p70. Compound **3** modulated the immune response by promoting anti-inflammatory cytokine IL-10 production, despite an inconsistent suppressive effect on pro-inflammatory cytokines. These results suggest that gamma-induced radiolysis is a useful strategy for enhancing the therapeutic potential of dietary phenolic compounds.

## 1. Introduction

*p*-Coumaric acid (4-hydroxycinnamic acid) is a predominant dietary phenolic compound ubiquitously distributed across the plant kingdom [1]. Structurally, it is characterized by a phenyl ring substituted with a hydroxyl group and an acrylic acid side chain, forming a reprehensive C6–C3 skeleton. This specific molecular arrangement promotes effective radical stabilization through electron donation, thereby conferring significant antioxidant properties [2]. Beyond its capacity as a free radical scavenger, *p*-coumaric acid exhibits a diverse pharmacological profile, including anti-microbial, anti-carcinogenic, and anti-diabetic effects [3]. Furthermore, it demonstrates immunomodulatory and anti-inflammatory activities by downregulating tumor necrosis factor-alpha (TNF-α) expression and regulating macrophage phagocytic functions [4]. Notably, this compound has been shown to be particularly effective in inhibiting the proliferation of and inducing apoptosis in colon cancer cells [5]. In addition, its metabolic benefits have been substantiated in diabetic models, where it enhances insulin sensitivity and maintains glycemic homeostasis [6]. Notably, it has been reported to mitigate lipopolysaccharide (LPS)-induced neurodegeneration and cognitive impairment by modulating oxidative stress, inflammation, and apoptosis [7].

Research into the radiolysis of organic compounds was initially driven by food safety assessment of irradiated products [8]. Fundamental studies have established that the radiolysis of water generates highly reactive species, including hydroxyl radical (∙OH), hydrogen atom (∙H), hydrogen gas (H_2_), hydrogen peroxide (H_2_O_2_), and hydrated electrons (e_aq_^−^), which can interact with phenolic scaffolds to yield structurally diverse derivatives [9]. While previous investigations primarily focused on component degradation, recent perspectives have redefined radiolysis as a strategic tool for intentional structural modification to improve biological functionalities and aqueous solubility compared to the parent compounds [10]. As a green chemistry approach, radiation technology enables the generation of derivative libraries through environmentally friendly, one-step reactions without the requirement of a toxic organic solvent [11]. Therefore, the systematic construction of irradiation-derived structural libraries from natural products offers a viable pathway for discovering lead candidates for functional medicines and nutraceuticals.

In the present study, we describe the structural elucidation of molecular derivatives generated from gamma irradiation of *p*-coumaric acid. Additionally, we evaluate their anti-inflammatory activities in LPS-stimulated RAW264.7 macrophages to determine the impact of structural modification on biological potency.

## 2. Results and Discussion

### 2.1. Isolation and Structure Elucidation of Radiolytic Derivatives ***1***–***3***

**Figure 1:**
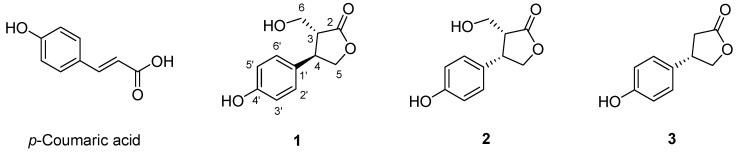
The chemical structures of **1**–**3** derived from gamma-irradiated *p*-coumaric acid.

Methanol solutions of *p*-coumaric acid were irradiated at doses of 20, 40, and 60 kGy to identify the degradation pattern of the original material and the resulting radiolysis products (Figure S1). The optimal irradiation dose was determined to be 60 kGy, as it resulted in the greatest reduction of the parent compound, *p*-coumaric acid, compared to the lower doses tested. The column chromatographic separation of the bulk radiolysis product of *p*-coumaric acid led to the isolation of three pure compounds each. Structurally modified compounds from *p*-coumaric acid were identified as two new structures, (3*R*,4*R*)-3-hydroxymethyl-4-(4-hydroxyphenyl)-dihydrofuran-2(3*H*)-one (**1**) and (3R*,4S*)-3-hydroxymethyl-4-(4-hydroxyphenyl)-dihydrofuran-2(3*H*)-one (**2**), and one known structure, (4*S*)-4-(4-hydroxyphenyl)-dihydrofuran-2(3*H*)-on (**3**), by analysis of their spectroscopic data. Their chemical structures are shown in Figure 1.

Compound **1** gave a molecular ion peak at *m*/*z* 231.0633 [M + Na]^+^ in the high-resolution electrospray ionization mass spectroscopy (HRESIMS), corresponding to the molecular formula of C_11_H_12_O_4_Na. The ^1^H and ^13^C nuclear magnetic resonance (NMR) spectra of **1** exhibited signals for a 1,4-disubstituted aromatic ring system at δ_H_ 6.78 (2H, d, *J* = 8.5 Hz, H-3′ and H-5′)/δ_C_ 127.2 (H-3′ and H-5′) and δ_H_ 7.19 (2H, d, *J* = 8.5 Hz, H-2′ and H-4′)/δ_C_ 114.6 (H-2′ and H-4′) (Table 1). In the ^1^H NMR spectrum of **1**, signals for two methine protons at δ_H_ 2.84 (1H, dt, *J* = 10.6, 3.3 Hz, H-3) and 3.84 (1H, td, *J* = 10.6, 8.4 Hz, H-4) and two sets of methylene protons at δ_H_ 3.62 (1H, dd, *J* = 11.4, 3.3 Hz, H-6), 3.95 (1H, dd, *J* = 11.4, 3.3 Hz, H-6), 4.16 (1H, dd, *J* = 10.6, 8.4 Hz, H-5a), and 4.54 (1H, t, *J* = 8.4 Hz, H-5b) indicated that **1** has a hydroxymethyl group attached to a γ-lactone, which was supported by ^1^H−^13^C HMBC NMR correlations of H-3/C-2, C-4, C-5, C-6, H-4/C-2, C-3, C-5, H-5/C-2, C-3, C-4, H-6/C-2, C-3, C-4. The HMBC correlations of H-2′ and H-6′/C-4, C-1′ and H-3′ and H-5′/C-1′, C-4′ established the elucidation of **1** as 3-hydroxymetyl-4-(4-hydroxyphenyl)-dihydrofuran-2-one. All of the ^1^H and ^13^C NMR assignment of **1** were confirmed by the aid of ^1^H−^1^H COSY, ^1^H−^1^H NOESY, ^1^H−^13^C HSQC, and ^1^H−^13^C HMBC NMR experiments on this molecule. The relative stereochemistry of **1** was suggested to be *trans*-oriented by comparing its coupling constant (*J*_3,4_ = 10.6 Hz) with the reported value of *anti*-3-ethyl-4-phenyldihydrofuran-2(3*H*)-one [12]. Compound **1** exhibited the electronic circular dichroism (ECD) spectrum identical to the reported (3*R*,4*S*) derivative, indicating that the C-3/C-4 substituents maintain a *trans*-relative configuration [13]. Despite this structural correspondence, the absolute stereochemistry of **1** was determined to be (3*R*,4*R*), as the ethylhydroxyl group at C-4 becomes the highest-priority substituent. Therefore, the structure of **1** was elucidated as (3*R*,4*R*)-3-hydroxymethyl-4-(4-hydroxyphenyl)-dihydrofuran-2(3*H*)-one.

The molecular ion peak of compound **2** at *m*/*z* 231.0634 [M + Na]^+^ corresponded to the chemical formula of C_11_H_12_O_4_Na, as confirmed by HRESIMS. Comparison of the ^1^H and ^13^C NMR spectral data of **2** with those of **1** indicated that these compounds have identical structures, differing in the chemical shifts and splitting patterns. The ^1^H NMR spectrum of 2 displayed signals at δ_H_ 3.05 (1H, ddd, *J* = 8.5, 7.3, 4.3 Hz, H-3), 3.34 (1H, dd, *J* = 10.8, 7.3 Hz, H-6), 3.60 (1H, dd, *J* = 10.8, 4.3 Hz, H-6), 3.84 (1H, ddd, *J* = 8.5, 6.6, 4.2 Hz, H-4), 4.49 (1H, dd, *J* = 8.9, 4.2 Hz, H-5a), and 4.57 (1H, dd, *J* = 8.9, 6.6 Hz, H-5b) for hydroxymethyl-dihydrofuran-2-one (Table 1). The key ^1^H−^13^C HMBC NMR correlations of H-3/C-2, C-4, C-5, C-6, C-2′, C-6′, H-4/C-2, C-3, C-5, H-5/C-2, C-3, C-4, H-1′, and H-6/C-2, C-3, C-4, C-2′, C-6′ were evidence that **2** was also 3-hydroxymetyl-4-(4-hydroxyphenyl)-dihydrofuran-2-one. Further detailed analysis of ^1^H−^1^H COSY, ^1^H−^13^C HSQC, and ^1^H−^13^C HMBC NMR data allowed unambiguous assignments for all of the ^1^H and ^13^C NMR signals of **2**. In contrast to **1**, compound **2** was assigned a *cis*-relative stereochemistry, as the vicinal coupling constants for H-3 and H-4 were smaller than those of **1** and were consistent with those of the reported compound *syn*-3-ethyl-4-phenyldihydrofuran-2(3*H*)-one [12]. In the ^1^H−^1^H NOESY spectra, a distinct correlation between H-3 and H-4 was observed for compound **2**, indicating a *cis* configuration, whereas this correlation was absent in compound **1**. However, reported CD data for analogs α,β-disubstituted γ-lactones with established absolute configurations are unavailable in the literature. Furthermore, the CD spectrum of **2** was distinct from that of **1**, exhibiting a positive Cotton effect at both 213 and 227 nm. Consequently, the absolute stereochemistry of **2** remains undetermined, and only its relative configuration has been elucidated. Therefore, the structure of **2** was elucidated as (3R*,4S*)-3-hydroxymethyl-4-(4-hydroxyphenyl)-dihydrofuran-2(3*H*)-one.

Compound **3** showed a molecular ion peak at *m*/*z* 201.0526 [M+Na]^+^, corresponding to an elemental formula C_10_H_10_O_3_Na through low-resolution ESIMS. The ^1^H and ^13^C NMR spectra of compound **3** were similar to those of rosmarinosin B [14], indicating that **3** is also a γ-butyrolactone derivative. The difference between the NMR spectroscopic data of **3** and rosmarinosin B were in the signals of the aromatic ring system, with compound **3** exhibiting the ^1^H and ^13^C NMR signals for a 1,4-disubstituted aromatic ring system at δ_H_ 6.76 (2H, d, *J* = 10.0 Hz, H-3′ and H-5′)/δ_C_ 116.8 (H-3′ and H-5′) and δ_H_ 7.71 (2H, d, *J* = 10.0 Hz, H-2′ and H-4′)/δ_C_ 129.1 (H-2′ and H-4′). The specific rotation value of **3** ([α]_D_^25^ +2.0) was opposite to that of rosmarinosin B ([α]_D_^25^–16.4), of which the reported absolute stereochemistry is determined to be *R* [14]. In addition, comparison with reported CD data determined the absolute configuration of **3** to be *S* [15]. Therefore, the structure of **3** was identified as (4*S*)-4-(4-hydroxyphenyl)-dihydrofuran-2(3*H*)-one. The *S* configuration of this structure is reported for the first time in this study.

**Figure 2:**
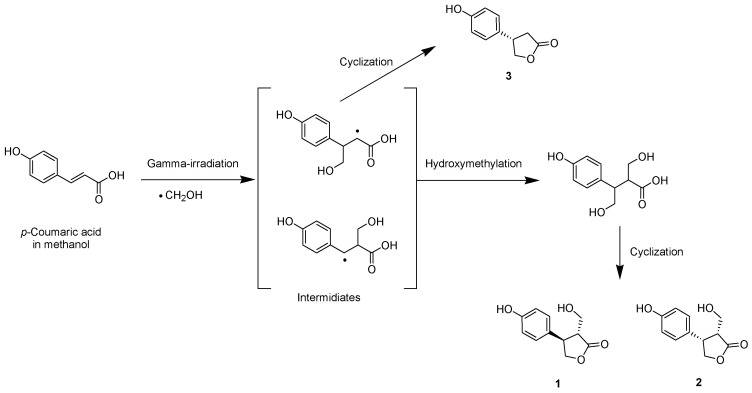
Proposed chemical transformation of *p*-coumaric acid by gamma irradiation.

The proposed chemical transformation of *p*-coumaric acid by gamma irradiation begins with the radiolysis of the methanol solvent, during which a highly reactive hydroxymethyl radical is generated (Figure 2). These species selectively target the exocyclic double bond of the parent compound to produce radical intermediates. Hydroxymethylation of the side chains forms radical intermediates, followed by intramolecular cyclization reactions to produce lactone derivatives, **1** and **2**. Alternatively, a direct cyclization of the radical intermediate leads to the formation of derivative **3**. This radical-mediated transformation demonstrates that γ-irradiation is an effective tool for generating structural diversity from natural phenolic acids, capable of producing both novel derivatives and known degradation products through addition, cyclization, and cleavage reactions [14,16].

### 2.2. Anti-Inflammatory Evaluation of Radiolytic Derivatives ***1***–***3***

**Figure 3:**
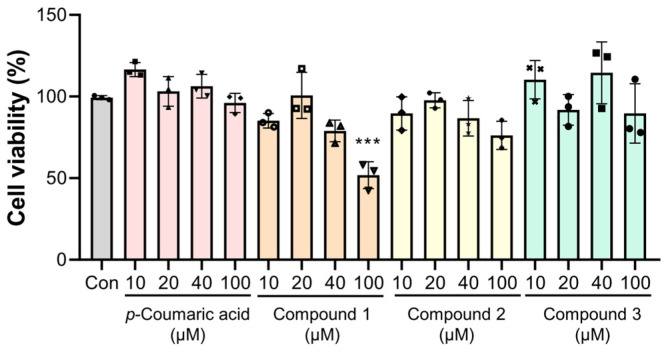
Effects of *p*-coumaric acid and its radiolytic derivatives (**1**–**3**) on the cell viability of RAW 264.7 cells. Cells were seeded in 96-well plates (5 × 10^4^ cells/well) and incubated overnight. Subsequently, the cells were treated with *p*-coumaric acid or compounds **1**–**3** at concentrations ranging from 10 to 100 μM and incubated for 24 h. The control group (Con) was treated with 0.25% DMSO, serving as the vehicle control. Cell viability was analyzed by MTT assay. Data are presented as means ± SD. Individual data points for each replicate (*n* = 3) are represented by the symbols (circles, squares, triangles, diamonds, and crosses). Statistical analysis was performed using one-way ANOVA followed by Tukey’s post hoc test. *** *p* < 0.001 represents statistical significance.

Prior to evaluating the anti-inflammatory potential, the cytotoxic profiles of *p*-coumaric acid and its radiolytic derivative were determined using the 3-(4,5-dimethylthiazol-2-yl)-2,5-diphenyltetrazolium bromide (MTT) assay in RAW 264.7 macrophages. Whiles *p*-coumaric acid and compounds **2** and **3** maintained cell viability across the tested range (10−100 μM), compound **1** exhibited cytotoxicity at 100 μM (Figure 3). The cytotoxicity of ibuprofen (IBF), utilized as a positive control, was assessed, and no significant decrease in cell viability was observed at concentrations up to 40 μM (Figure S2). Consequently, a maximum dose of 40 μM was selected for subsequent cytokine quantification to ensure that the observed inhibitory effects were not attributable to cell death, thereby establishing the optimal concentration range for evaluating anti-inflammatory potency.

**Figure 4:**
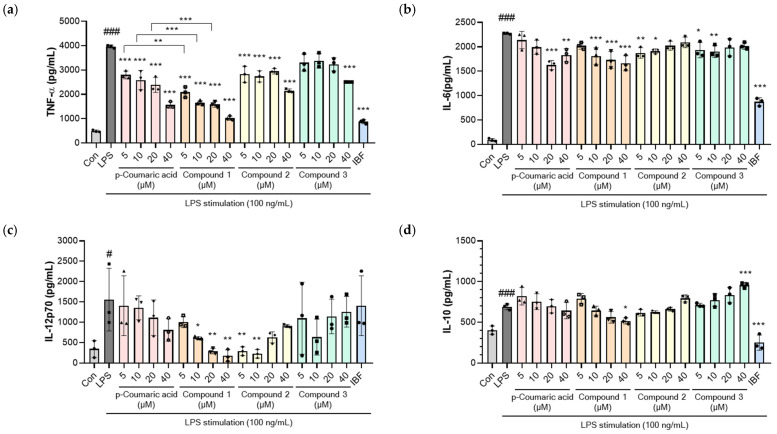
Effects of *p*-coumaric acid and its radiolytic derivatives (**1**–**3**) on cytokine production in LPS-stimulated RAW 264.7 cells. Cells were seeded in 96-well plates (5 × 10^4^ cells/well) and incubated overnight. Subsequently, the cells were treated with *p*-coumaric acid or compounds **1**–**3** at concentrations ranging from 5 to 40 μM and incubated for 8 h under LPS stimulation (100 ng/mL). The control group (Con) and the LPS-only group were treated with 0.25% DMSO, serving as the vehicle control. Ibuprofen (IBF) is used as positive control (40 μM). The levels of pro-inflammatory cytokines, including TNF-α (**a**), IL-6 (**b**), and IL-12p70 (**c**) and the anti-inflammatory cytokine (IL-10) (**d**) in the culture supernatants were analyzed using commercial ELISA kits. Data are presented as means ± SD. Individual data points for each replicate (*n* = 3) are represented by the symbols (circles, squares, triangles, and diamonds). Statistical analysis was performed using one-way ANOVA followed by Tukey’s post hoc test. ^#^ *p* < 0.05 and ^###^ *p* < 0.001 represent statistical significance between the control group and the LPS-only group. * *p* < 0.05, ** *p* < 0.01, and *** *p* < 0.001 represent statistical significance compared to the LPS-only group.

As shown in Figure 3, LPS (100 ng/mL) stimulation induced a robust inflammatory response, characterized by increased secretion of TNF-α, interleukin-6 (IL-6), interleukin-12p70 (IL-12p70), and interleukin-10 (IL-10). The parent compound, *p*-coumaric acid, reduced TNF-α levels at all concentrations (Figure 4a) and suppressed IL-6 production at doses above 20 μM (Figure 4b), while showing no significant effects on IL-12p70 or IL-10 levels (Figure 4b,c). In contrast, the radiolytic derivatives exhibited distinct immunomodulatory effects. Notably, compound **1** showed significantly more potent inhibitory activity against TNF-α secretion compared to the parent compound at identical concentrations (5–40 μM) (Figure 4a). Furthermore, compound **1** significantly attenuated IL-6 and IL-12p70 levels within the 10−40 μM range (Figure 4b,c). Compound **2** also demonstrated inhibitory activity, reducing TNF-α at all doses and suppressing IL-6 and IL-12p70, particularly at lower concentrations (5−10 μM). Meanwhile, compound **3** displayed a different trend; while it inhibited TNF-α only at 40 μM and IL-6 at 5−10 μM, it markedly upregulated the secretion of the anti-inflammatory cytokine IL-10. IBF, a representative non-steroidal anti-inflammatory drug (NSAID), was utilized as a well-known positive control at a concentration of 40 μM. IBF reduced TNF-α, IL-6, and IL-10, but did not affect IL-12p70 levels. Notably, compound **1** demonstrated more potent inhibitory effects than IBF on pro-inflammatory signaling, especially regarding IL-12p70 production.

The differential cytokine regulation observed among the radiolytic derivatives suggests distinct therapeutic mechanisms. While TNF-α and IL-6 are primary mediators of acute inflammation, IL-12p70 serves as a critical Th1-polarizing cytokine bridging innate and adaptive immunity [17,18]. Although these cytokines are primarily regulated by the TLR-4-MyD88-NF-kB signaling pathway, their specific regulatory mechanisms vary. TNF-α is regulated at both the transcriptional and translational levels, leading to its rapid induction immediately following LPS stimulation. In contrast, IL-6 is primarily regulated at the transcriptional level and exhibits a characteristically delayed response; this mechanistic differences may explain the relative insensitivity of IL-6 to specific kinase inhibitors that effectively suppress TNF-alpha [19]. Recent studies suggest that the IRAK4 scaffold functions as a pivotal integrator of TLR4-driven MYD88 and TRIF pathways to activate TRAF6. This signaling hub facilitates the selective regulation of pro-inflammatory mediators: specifically, the recruitment of RelA (p65) is indispensable for TNF-α expression, whereas c-Rel uniquely mediates the production of IL-12 [20]. The superior inhibitory potency of compound **1** against both TNF-alpha and IL-12p70, compared to the standard NSAID IBF, suggests its potential to selectively interfere with this IRAK4-mediated TRAF6 activation node or its specific downstream NF-kappaB subunits. Further studies, including detailed mechanistic investigations and in vivo efficacy and safety evaluations, are needed to fully elucidate the therapeutic potential and clinical applicability of these radiolytic derivatives.

A pivotal finding of this study is the profound influence of stereochemistry on pharmacological efficacy. Although compounds **1** and **2** share an identical planar structure, they differ in their spatial arrangement, which dictates their topographic complexity and target engagement. Such stereoselective bioactivity is well-documented in medicinal chemistry. For instance, the natural form of tetrahydrolipstatin (THL) exhibits a nearly 19-fold-higher IC_50_ for lipase inhibition compared to its enantiomer, as its specific stereoconfiguration is geometrically optimized for the enzyme’s active site [21]. Similarly, the absolute configuration of both stereocenters in atorvastatin must be (*R*,*R*) to maintain high activity, with other diastereomers exhibiting significantly diminished efficacy [22]. Consistent with these precedents, the enhanced anti-inflammatory activity of compound **1** (3*R*,4*R*) compared to its *cis*-derivative (compound **2**) is likely attributed to the precise stereochemical fit of its *trans*-configuration within the chiral binding pockets of its molecular targets.

## 3. Materials and Methods

### 3.1. General Procedures

Gamma-irradiation was carried out using a cobalt-60 experimental irradiator (150 TBq capacity; AECL, Nordion International Co., Ltd., Ottawa, ON, Canada), with a source intensity of approximately 320 kCi and a dose rate of 10 kGy/h. NMR spectra were obtained from a Varian NMR System 500 MHz (^1^H NMR at 500 MHz, ^13^C NMR at 125 MHz, Varian, Palo Alto, CA, USA). HRESIIMS was performed on a Waters SYNAPT G2 mass spectrometer (Waters Corporation, Milford, CT, USA). Optical rotation was obtained using a JASCO P-2000 polarimeter (JASCO, Tokyo, Japan). CD spectra were acquired on a JASCO J-715 spectrometer (JASCO). Column chromatography (CC) was performed using YMC gel ODS-A (50 μm; YMC Co., Kyoto, Japan) and Sephadex LH-20 (25–100 μm; GE Healthcare Biosciences AB, Uppsala, Sweden) gel columns. Analytical high-performance liquid chromatography coupled with photodiode array detection (HPLC–PDA) was carried out on an Agilent HPLC 1200 system and PDA 1200 Infinity series (Agilent Technologies, Palo Alto, CA, USA), equipped with YMC-Pack ODS A-302 columns (4.6 × 150 mm, 5 μm; YMC Co.). Semi-preparative HPLC was performed using a Waters 1525 Binary HPLC Pump system and Waters 996 Photodiode Array Detector (Waters Corporation), equipped with a YMC-Pack ODS-AQ column (10 × 250 mm; 5 μm, YMC Co.).

### 3.2. Sample Preparation and Analytical HPLC-PDA Experiments

*p*-Coumaric acid was purchased from Sigma-Aldrich (St. Louis, MO, USA). An amount of 10 mg of *p*-coumaric acid was dissolved in 10 mL of methanol and subjected to γ-irradiation at absorbed doses of 20, 40, and 60 kGy. The γ-irradiated samples were analyzed using HPLC, equipped with the analytical column. Gradient elution consisted of 0.5% formic acid in water (A) and acetonitrile (B) and eluted 5% B to 95% B for 30 min. Samples were injected with 10 μL at a flow rate of 1 mL/min. Chromatograms were acquired at 280 nm using a PDA detector. To obtain the bulk radiolysis product, 1 g of *p*-coumaric acid was dissolved in 1 L of methanol and irradiated at 60 kGy. After gamma irradiation, the solvent was removed using a rotary evaporator (FDU-1200, EYELA, Tokyo, Japan) under reduced pressure at 40 °C, and the resulting residue was subsequently lyophilized using a freeze-dryer (FD-8508, Ilshin BioBase, Dongducheon-si, Republic of Korea) at −80 °C under a vacuum of less than 10 mTorr for 24 h to ensure complete removal of residual moisture.

### 3.3. Isolation of the Radiolysis Products

The radiolysis product of *p*-coumaric acid was subjected to Sephadex LH-20 CC using the solvent system of 100% methanol to yield seven fractions (F01–F07). Fraction F02 (120 mg) was subjected to reverse phase CC using methanol/water (1:1 to 1:0, *v*/*v*) as the gradient solvent system, yielding seven sub-fractions (F0201–F0207). Sub-fraction F0203 (38 mg) was subjected to semi-preparative HPLC using methanol/water (1:1, *v*/*v*; 2 mL/min) as the isocratic solvent system to afford compound **3** (21.5 mg, *t*_R_ 9.7 min). Sub-fraction F0201 was purified using the semi-preparative column with methanol/water (1:1, *v*/*v*; 2 mL/min) as the isocratic solvent system to obtain compound **1** (13.6 mg, *t*_R_ 12.5 min). Sub-fraction F0202 was purified using the semi-preparative column with methanol/water (1:4, *v*/*v*; 2 mL/min) as the isocratic solvent system to yield compound **2** (15.2 mg, *t*_R_ 38.7 min).

(3*R*,4*R*)-3-hydroxymethyl-4-(4-hydroxyphenyl)-dihydrofuran-2(3*H*)-one (**1**): Colorless solid, [α]_D_^25^ +2.9 (*c* 1.0, methanol); UV *λ*_max_ nm (log *ε*): 236, 275 nm; CD (methanol) Δ*ε* (nm): 214 (+10.2), 229 (−9.98); ^1^H and ^13^C NMR, see Table 1; HRESIMS *m*/*z* 231.0633 [M + Na]^+^ (calculated for C_11_H_12_O_4_Na, 231.0633) (Figures S3–S10).

(3R*,4S*)-3-hydroxymethyl-4-(4-hydroxyphenyl)-dihydrofuran-2(3*H*)-one (**2**): Colorless solid, [α]_D_^25^ +12.5 (*c* 1.0, methanol); UV *λ*_max_ nm: 232, 276 nm; CD (methanol) Δ*ε* (nm): 213 (+0.36), 227 (+1.98), 257 (+0.42) nm; ^1^H and ^13^C NMR, see Table 1; HRESIMS *m*/*z* 231.0634 [M + Na]^+^ (calculated for C_11_H_12_O_4_Na, 231.0633) (Figures S11–S18).

(4*S*)-4-(4-hydroxyphenyl)-dihydrofuran-2(3*H*)-one (**3**): Colorless solid, [α]_D_^25^ +2.0 (*c* 1.0, methanol); UV *λ*_max_ nm (log *ε*): 232, 279 nm; ^1^H and ^13^C NMR, see Table 1; HRESIMS *m*/*z* 201.0526 [M + Na]^+^ (calculated for C_10_H_10_O_3_Na, 201.0528) (Figures S19–S24).

### 3.4. Cell Culture and Cell Viability Assay

The RAW 264.7 murine macrophage cell line was purchased from the Korean Cell Line Bank (KCLB, Seoul, Republic of Korea). The cells were maintained in high glucose Dulbecco’s Modified Eagle Medium (DMEM) supplemented with 10% fetal bovine serum (FBS; Biowest, Nuaillé, France) and 1% penicillin–streptomycin (Gibco, Grand Island, NY, USA). The culture was incubated at 37 °C in a humidified atmosphere containing 5% CO_2_. For the cell viability assay, the cells were seeded in 96-well plates at a density of 5 × 10^4^ cells per well and allowed to adhere overnight. *p*-Coumaric acid and its radiolytic derivatives (**1**−**3**) were dissolved in dimethyl sulfoxide (DMSO) and stored as stock solutions at −20 °C. For experimental treatments, these stocks were further diluted in the culture medium to ensure that the final concentration of DMSO remained below 0.25%, thereby minimizing potential solvent-induced cytotoxicity. The cells were treated with various concentrations of the parent compound and its derivatives and subsequently incubated for 24 h at 37 °C in a humidified atmosphere containing 5% CO_2_. The control group was treated with 0.25% DMSO, serving as the vehicle control. Following the incubation period, cell viability was evaluated using MTT assay (Sigma-Aldrich). The cells were incubated with 0.5 mg/mL of the MTT reagent for 1 h. After the staining process, the resulting formazan crystals were solubilized in DMSO. The absorbance of each well was measured at 570 nm using a microplate reader to determine the relative cell viability compared to the control group. The relative cell viability was calculated as a percentage of the absorbance observed in the vehicle control group.

### 3.5. Measurement of Cytokines Levels by ELISA

To quantify the production of cytokines, RAW264.7 cells were seeded in 96-well plates at a density of 5 × 10^4^ cells per well and incubated overnight for adherence. *p*-Coumaric acid and its radiolytic derivatives (**1**–**3**) were treated at doses from 5 to 40 μM in the presence of 100 ng/mL of lipopolysaccharide (LPS; InvivoGen, San Diego, CA, USA) stimulation. After 8 h of incubation, the culture supernatants were collected and stored at −20 °C for subsequent analysis. The concentrations of TNF-alpha, IL-6, IL-12p70, and IL-10 in the supernatants were determined using commercial enzyme-linked immunosorbent assay (ELISA) kits (BD Biosciences, San Jose, CA, USA) according to the manufacturer’s instructions. The absorbance was measured using a microplate reader, and the cytokine concentrations were calculated based on the standard curves generated for each protein.

### 3.6. Statistical Analysis

All experimental results are presented as the mean ± standard deviation (SD). Statistical significance between the experimental groups was determined by a one-way analysis of variance (ANOVA) followed by Tukey’s post hoc test for multiple comparisons. All statistical analyses were performed using GraphPad Prism software (version 8.0; GraphPad Software, San Diego, CA, USA).

## 4. Conclusions

In summary, gamma irradiation was successfully employed for the structural modification of *p*-coumaric acid. This radiolysis process yields two novel derivatives, **1** and **2**, along with **3**, a new isomer of the known compound. Our results demonstrate that ionizing radiation can efficiently modify phenolic scaffolds to produce derivatives with enhanced pharmacological properties. Compound **1** exhibited a superior anti-inflammatory effect compared to the parent compound, *p*-coumaric acid, characterized by the significant inhibition of TNF-alpha, IL-6, and IL-12p70. The comparative evaluation of compounds **1** and **2** indicates that stereochemistry is a critical factor in biological efficacy; the *trans*-configuration of compound **1** likely provides a more precise fit with the chiral binding pockets of inflammatory targets. Additionally, the unique IL-10 upregulation by compound **3** suggests a distinct immunomodulatory mechanism. These findings confirm that radiation technology is a viable, green chemistry-based strategy for constructing structural libraries from natural products. This approach facilitated the identification of high-potency lead candidates for the development of nutraceuticals and anti-inflammatory therapeutics.

## Figures and Tables

**Table 1:** The ^1^H and ^13^C NMR data of compounds **1**–**3** (methanol-*d*_4_, 500 MHz for ^1^H NMR, 125 MHz for ^13^C NMR).

	1		2		3	
Position	*δ*_H_, Mult (*J* in Hz)	*δ*_C_	*δ*_H_, Mult (*J* in Hz)	*δ*_C_	*δ*_H_, Mult (*J* in Hz)	*δ*_C_
2	–	177.4	–	178.4	–	179.8
3	2.84 (dt, 10.6, 3.3)	48.9	3.05 (ddd, 8.5, 7.3, 4.3)	47.3	2.64 (dd, 18.7, 10.0) 2.84 (dd, 18.7, 10.0)	36.9
4	3.84 (td, 10.6, 8.4)	41.3	3.84 (ddd, 8.5, 6.6, 4.2)	43.2	3.73 (t, 10.0)	41.9
5	4.16 (dd, 10.6, 8.4) 4.54 (t, 8.4)	71.7	4.49 (dd, 8.9, 4.2) 4.57 (dd, 8.9, 6.6)	73.2	4.20 (t, 10.0) 4.61 (t, 10.0)	76.1
6	3.62 (dd, 11.4, 3.3) 3.95 (dd, 11.4, 3.3)	56.5	3.34 (dd, 10.8, 7.3) 3.60 (10.8, 4.3)	58.3	–	131.9
1′	–	128.1	–	128.4	–	–
2′	7.19 (d, 8.5)	127.2	7.08 (d, 8.6)	128.8	7.71 (br d, 10.0)	129.1
3′	6.78 (d, 8.5)	114.6	6.76 (d, 8.6)	115.2	6.76 (br d, 10.0)	116.8
4′	–	155.9	–	156.6	–	158.0
5′	6.78 (d, 8.5)	114.6	6.76 (d, 8.6)	115.2	6.76 (br d, 10.0)	116.8
6′	7.19 (d, 8.5)	127.2	7.08 (d, 8.6)	128.8	7.71 (br d, 10.0)	129.1

## Data Availability

The data presented in this study are available upon request from the corresponding authors.

## Supplementary Materials

The following supporting information can be downloaded at: https://www.mdpi.com/article/10.3390/molecules31101630/s1, Figure S1: HPLC profiles of *p*-coumaric acid irradiated with the following doses: 20, 40, and 60 kGy; Figure S2: Effects of ibuprofen (IBF) on the cell viability of RAW 264.7 cells; Figure S3: HRESIMS spectrum of compound **1**; Figure S4: ^1^H NMR spectrum of compound **1**; Figure S5: ^13^C NMR spectrum of compound **1**; Figure S6: ^1^H−^1^H COSY spectrum of compound **1**; Figure S7: ^1^H−^1^H NOESY spectrum of compound **1**; Figure S8: ^1^H−^13^C HSQC spectrum of compound **1**; Figure S9: ^1^H−^13^C HMBC spectrum of compound **1**; Figure S10: ECD spectrum of compound **1**; Figure S11: HRESIMS spectrum of compound **2**; Figure S12: ^1^H NMR spectrum of compound **2**; Figure S13: ^13^C NMR spectrum of compound **2**; Figure S14: ^1^H−^1^H COSY spectrum of compound **2**; Figure S15: ^1^H−^1^H NOESY spectrum of compound **2**; Figure S16: ^1^H−^13^C HSQC spectrum of compound **2**; Figure S17: ^1^H−^13^C HMBC spectrum of compound **2**; Figure S18: ECD spectrum of compound **2**; Figure S19: HRESIMS spectrum of compound **3**; Figure S20: ^1^H NMR spectrum of compound **3**; Figure S21: ^13^C NMR spectrum of compound **3**; Figure S22: ^1^H−^1^H COSY spectrum of compound **2**; Figure S23: ^1^H−^13^C HSQC spectrum of compound **3**; Figure S24: ^1^H−^13^C HMBC spectrum of compound **3**.
