# Supplementary material for: Radiolysis Derivatives from p-Coumaric Acid via Gamma Irradiation and Their Anti-Inflammatory Activities
Source: Molecules. 2026 May 12;31(10):1630. doi: 10.3390/molecules31101630 (PMC13209710; doi:10.3390/molecules31101630)
Supplement: Supplementary file 1 [file molecules-31-01630-s001.zip › molecules-4290013-supplementary.pptx]

## Slide 1
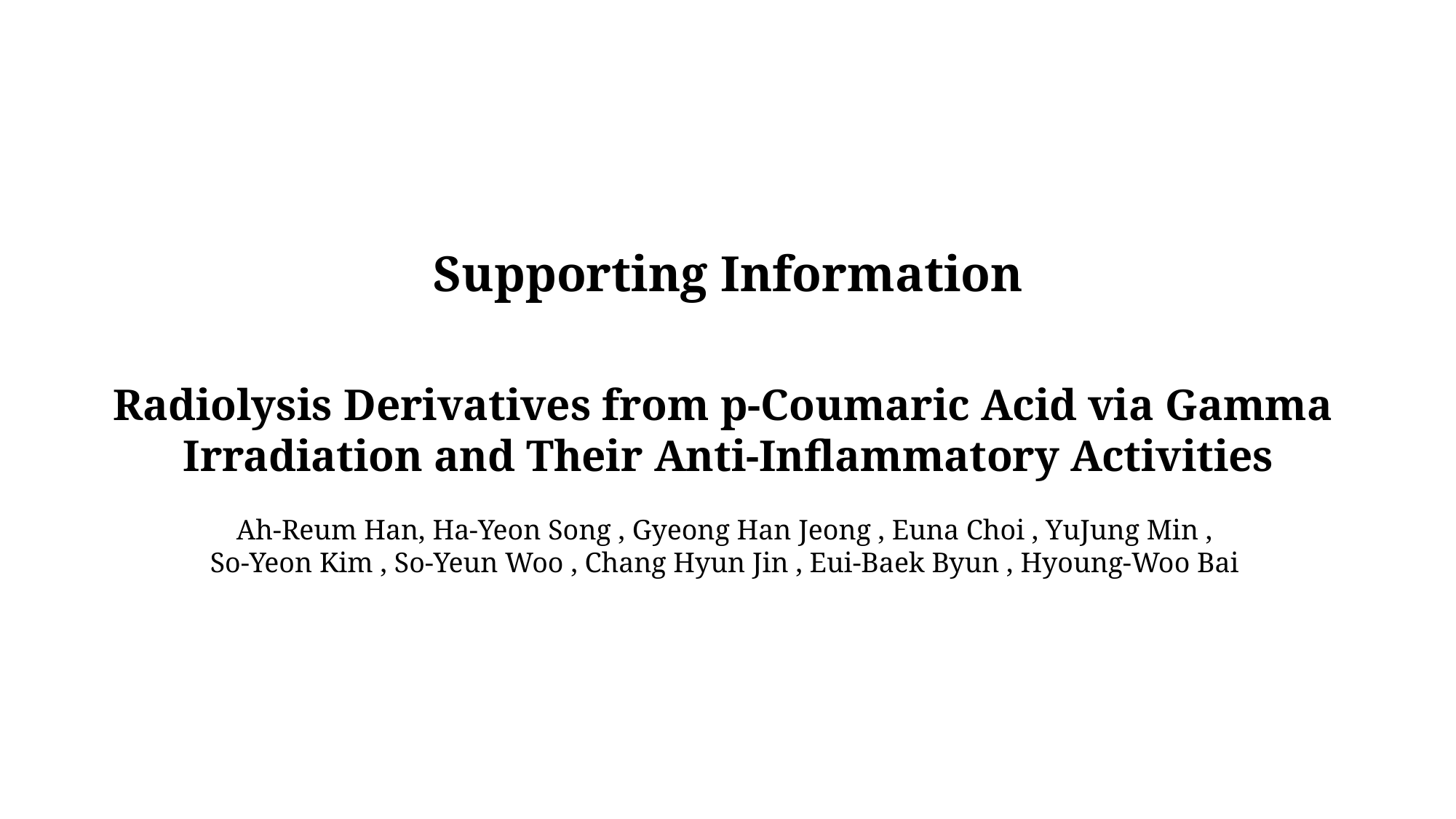

Supporting Information
Radiolysis Derivatives from p-Coumaric Acid via Gamma
Irradiation and Their Anti-Inflammatory Activities
Ah-Reum Han, Ha-Yeon Song , Gyeong Han Jeong , Euna Choi , YuJung Min ,
So-Yeon Kim , So-Yeun Woo , Chang Hyun Jin , Eui-Baek Byun , Hyoung-Woo Bai

## Slide 2
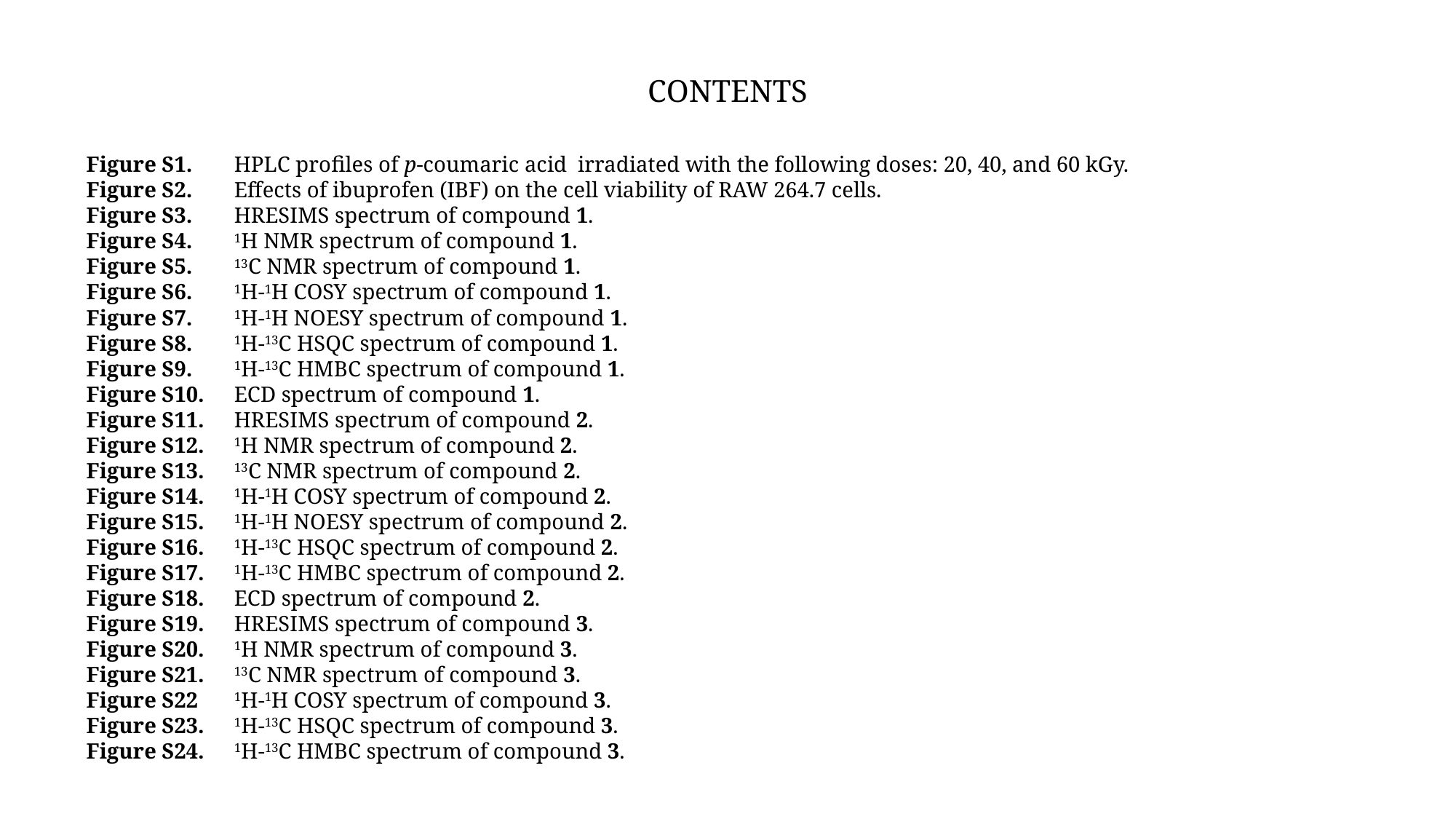

CONTENTS
| Figure S1. | HPLC profiles of p-coumaric acid irradiated with the following doses: 20, 40, and 60 kGy. |
| --- | --- |
| Figure S2. | Effects of ibuprofen (IBF) on the cell viability of RAW 264.7 cells. |
| Figure S3. | HRESIMS spectrum of compound 1. |
| Figure S4. | 1H NMR spectrum of compound 1. |
| Figure S5. | 13C NMR spectrum of compound 1. |
| Figure S6. | 1H-1H COSY spectrum of compound 1. |
| Figure S7. | 1H-1H NOESY spectrum of compound 1. |
| Figure S8. | 1H-13C HSQC spectrum of compound 1. |
| Figure S9. | 1H-13C HMBC spectrum of compound 1. |
| Figure S10. | ECD spectrum of compound 1. |
| Figure S11. | HRESIMS spectrum of compound 2. |
| Figure S12. | 1H NMR spectrum of compound 2. |
| Figure S13. | 13C NMR spectrum of compound 2. |
| Figure S14. | 1H-1H COSY spectrum of compound 2. |
| Figure S15. | 1H-1H NOESY spectrum of compound 2. |
| Figure S16. | 1H-13C HSQC spectrum of compound 2. |
| Figure S17. | 1H-13C HMBC spectrum of compound 2. |
| Figure S18. | ECD spectrum of compound 2. |
| Figure S19. | HRESIMS spectrum of compound 3. |
| Figure S20. | 1H NMR spectrum of compound 3. |
| Figure S21. | 13C NMR spectrum of compound 3. |
| Figure S22 | 1H-1H COSY spectrum of compound 3. |
| Figure S23. | 1H-13C HSQC spectrum of compound 3. |
| Figure S24. | 1H-13C HMBC spectrum of compound 3. |

## Slide 3
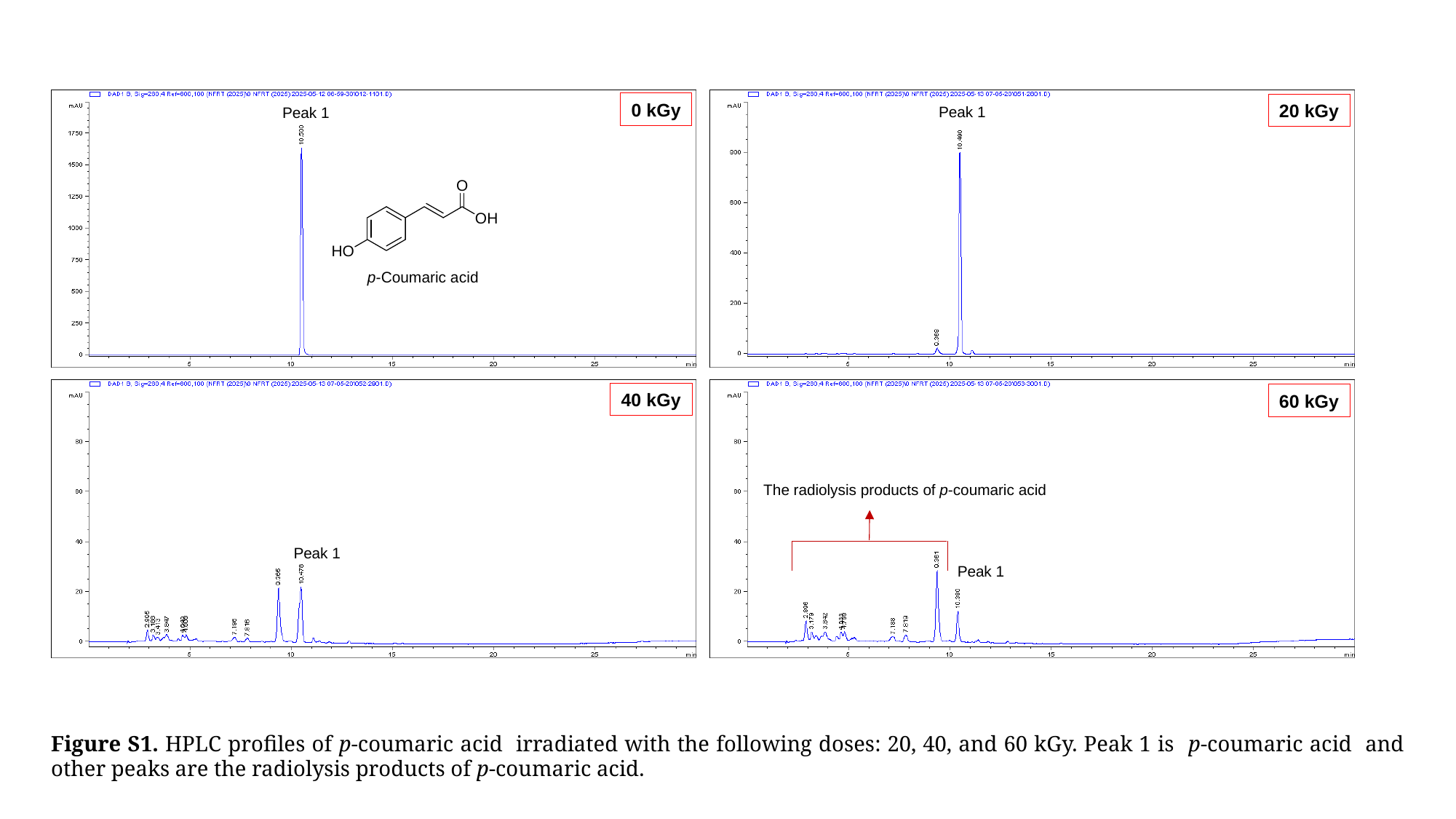

0 kGy
20 kGy
40 kGy
60 kGy
The radiolysis products of p-coumaric acid
Peak 1
Peak 1
Peak 1
Peak 1
Figure S1. HPLC profiles of p-coumaric acid irradiated with the following doses: 20, 40, and 60 kGy. Peak 1 is p-coumaric acid and other peaks are the radiolysis products of p-coumaric acid.

## Slide 4
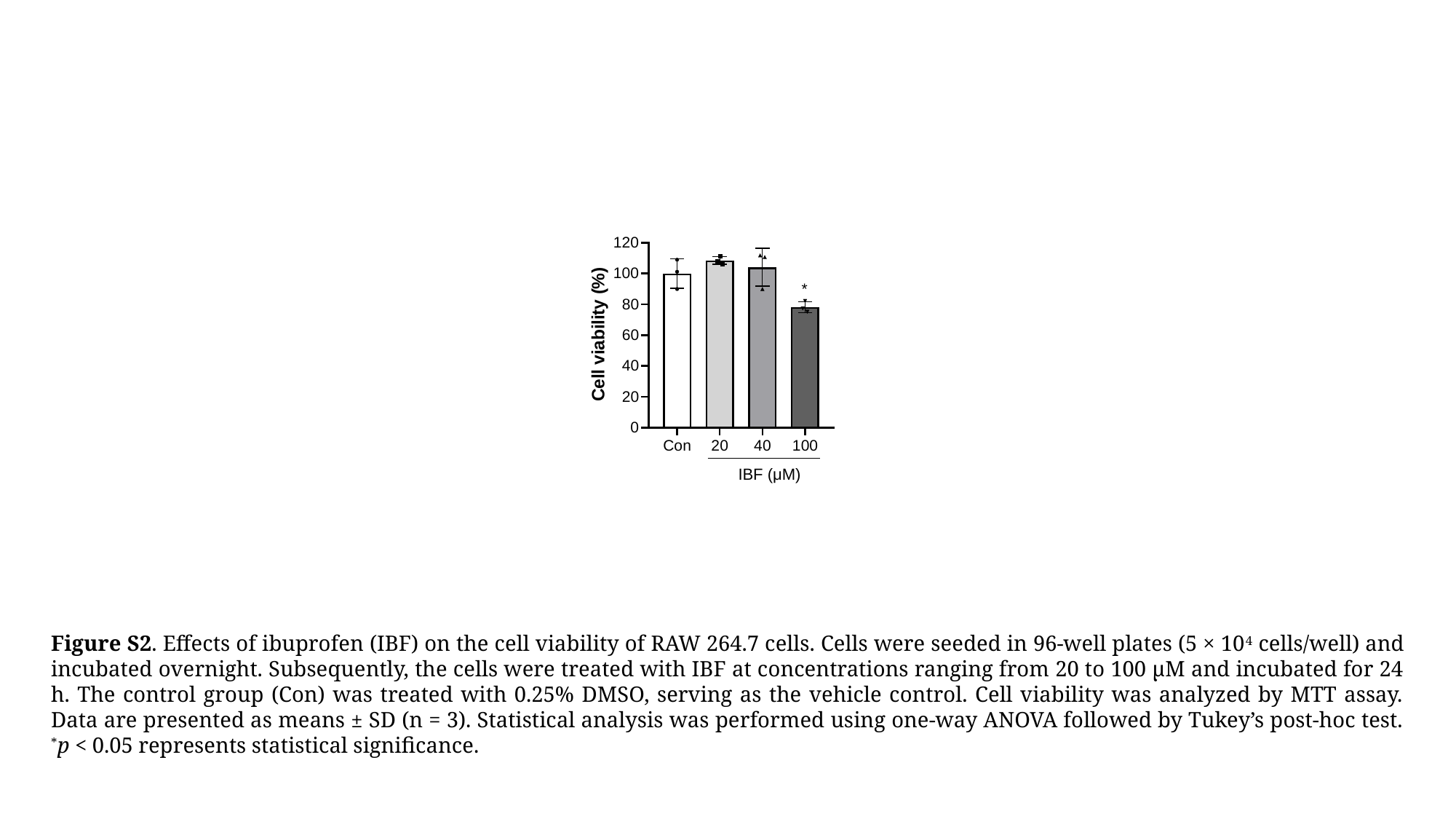

IBF (μM)
Figure S2. Effects of ibuprofen (IBF) on the cell viability of RAW 264.7 cells. Cells were seeded in 96-well plates (5 × 10^4^ cells/well) and incubated overnight. Subsequently, the cells were treated with IBF at concentrations ranging from 20 to 100 μM and incubated for 24 h. The control group (Con) was treated with 0.25% DMSO, serving as the vehicle control. Cell viability was analyzed by MTT assay. Data are presented as means ± SD (n = 3). Statistical analysis was performed using one-way ANOVA followed by Tukey’s post-hoc test. *p < 0.05 represents statistical significance.

## Slide 5
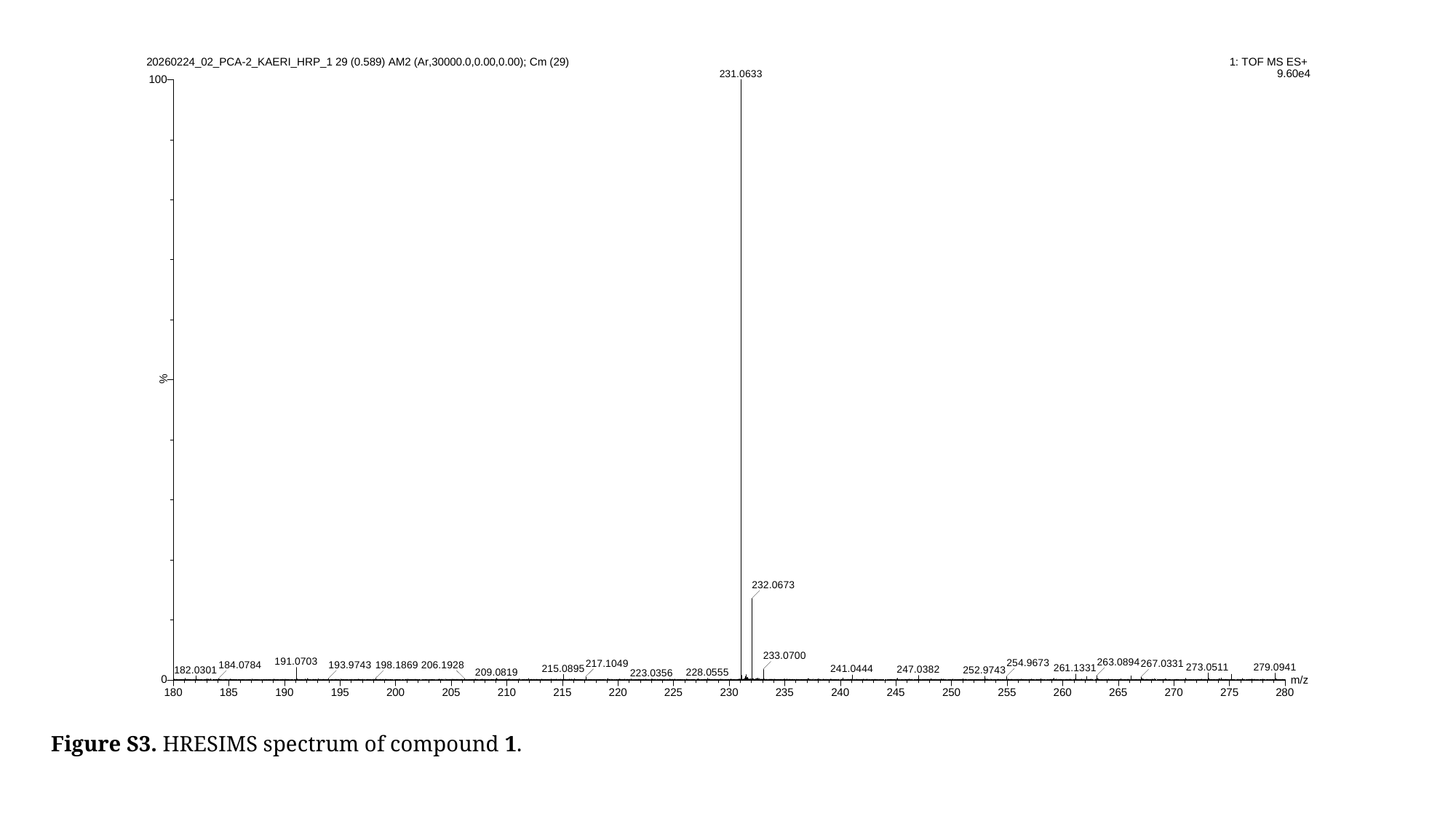

Figure S3. HRESIMS spectrum of compound 1.

## Slide 6
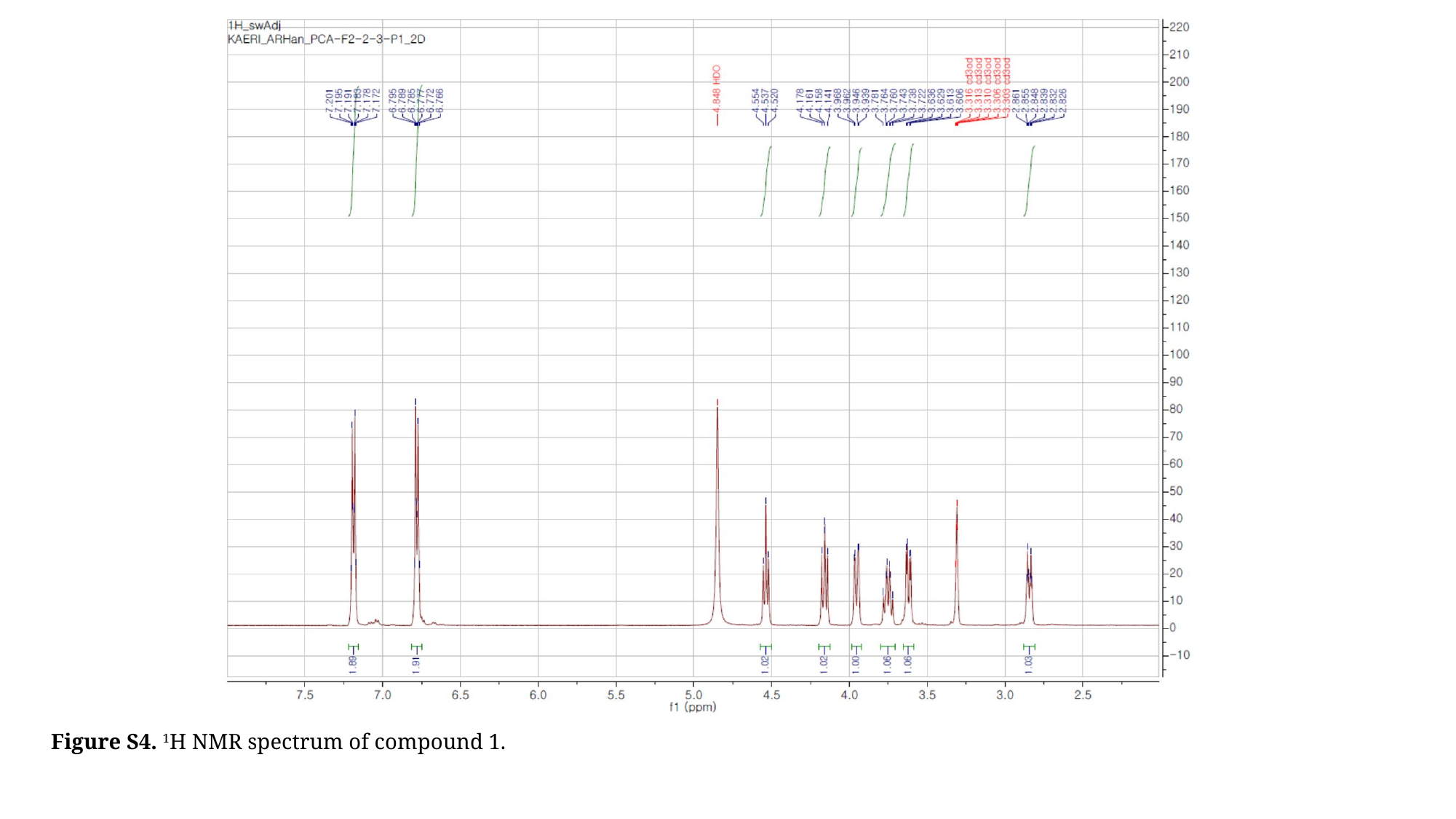

Figure S4. 1H NMR spectrum of compound 1.

## Slide 7
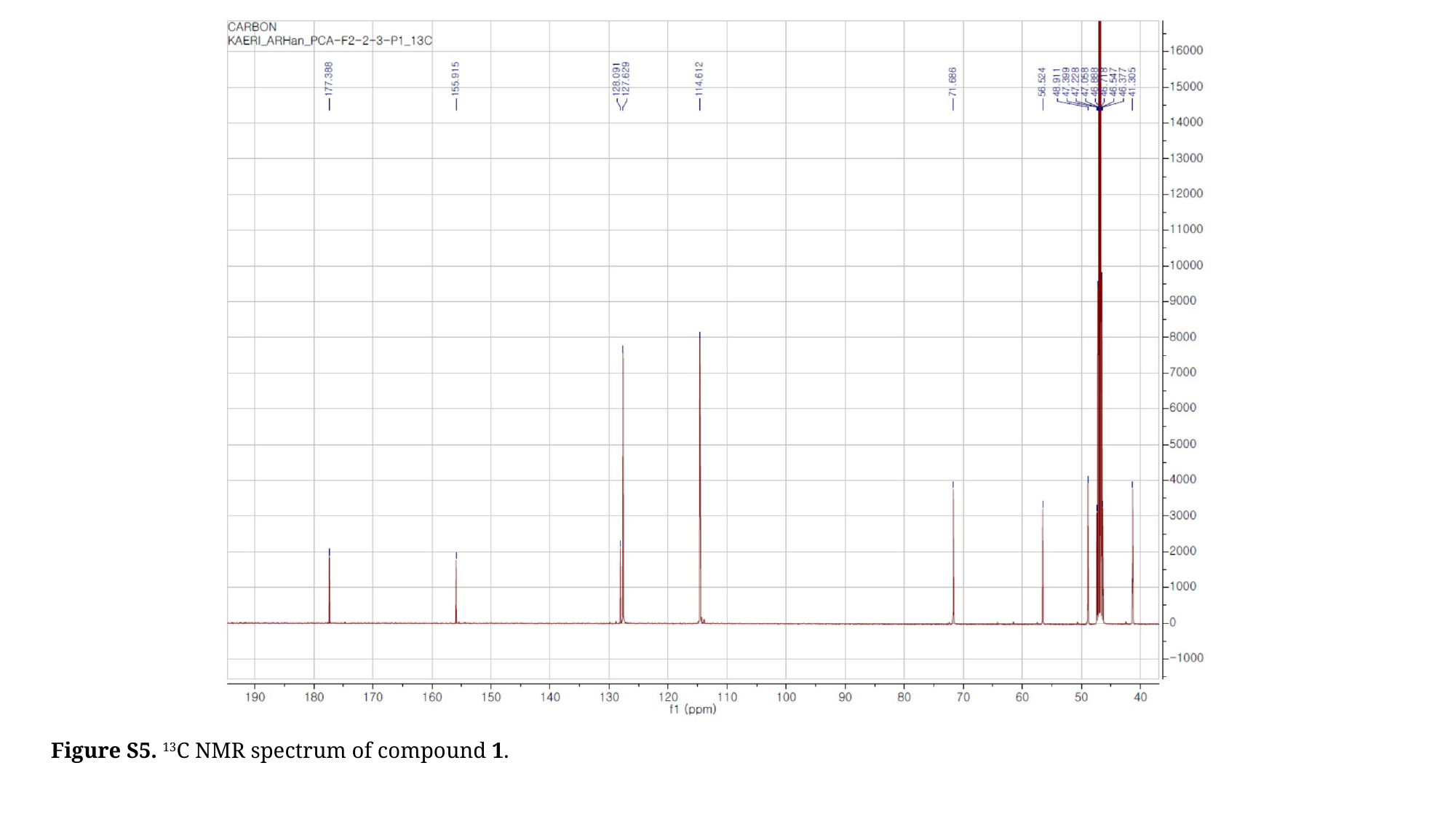

Figure S5. 13C NMR spectrum of compound 1.

## Slide 8
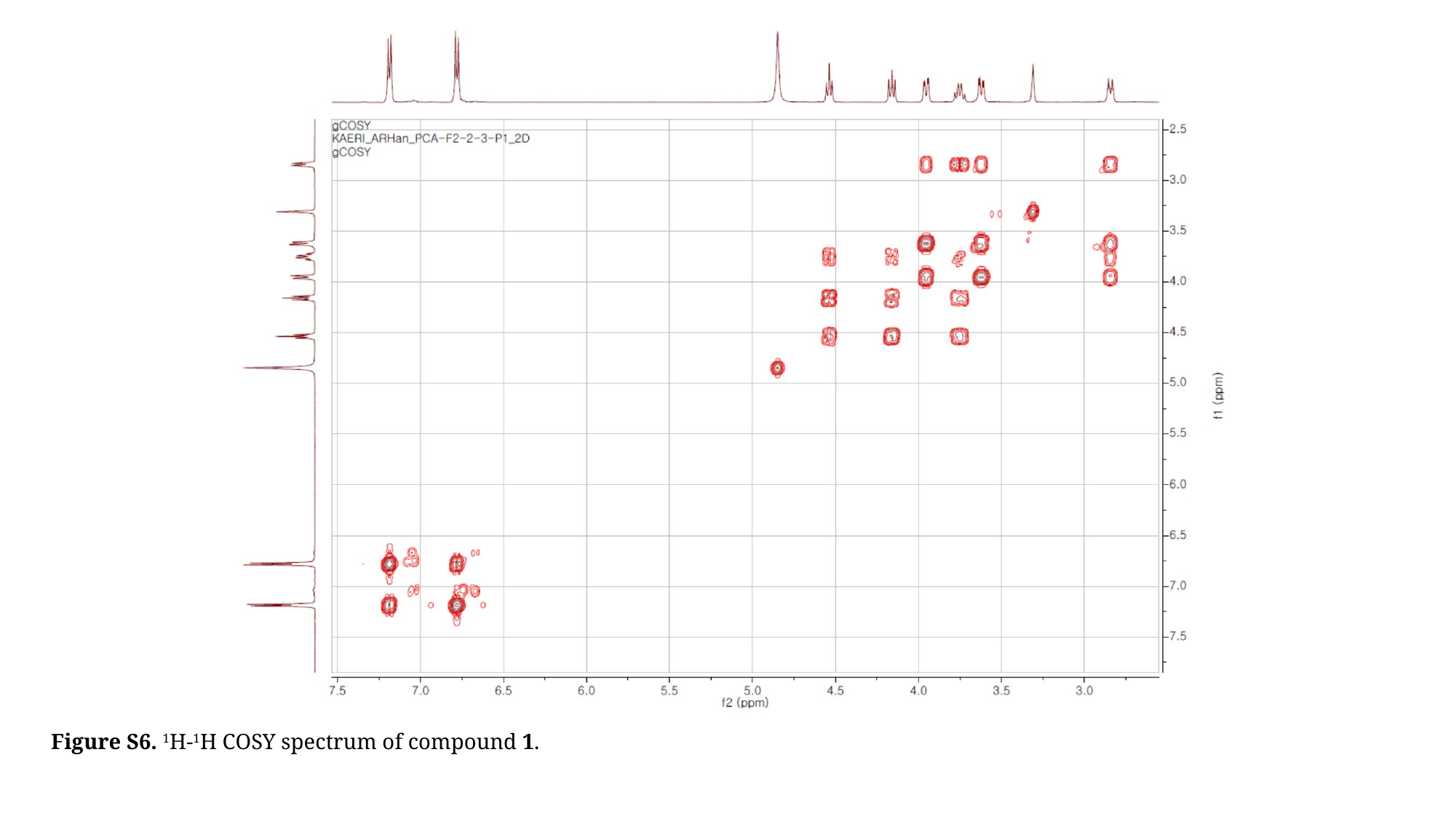

Figure S6. 1H-1H COSY spectrum of compound 1.

## Slide 9
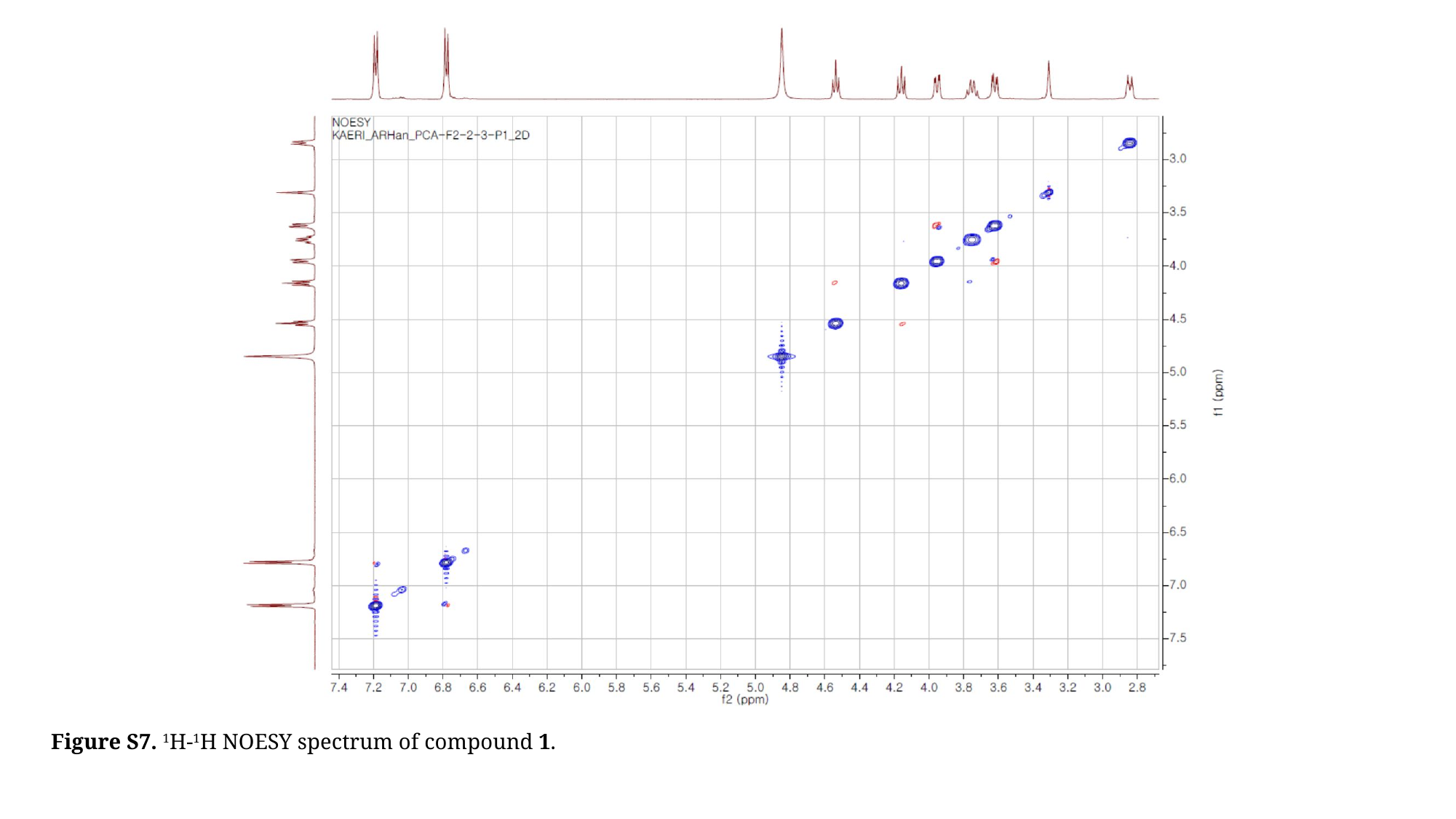

Figure S7. 1H-1H NOESY spectrum of compound 1.

## Slide 10
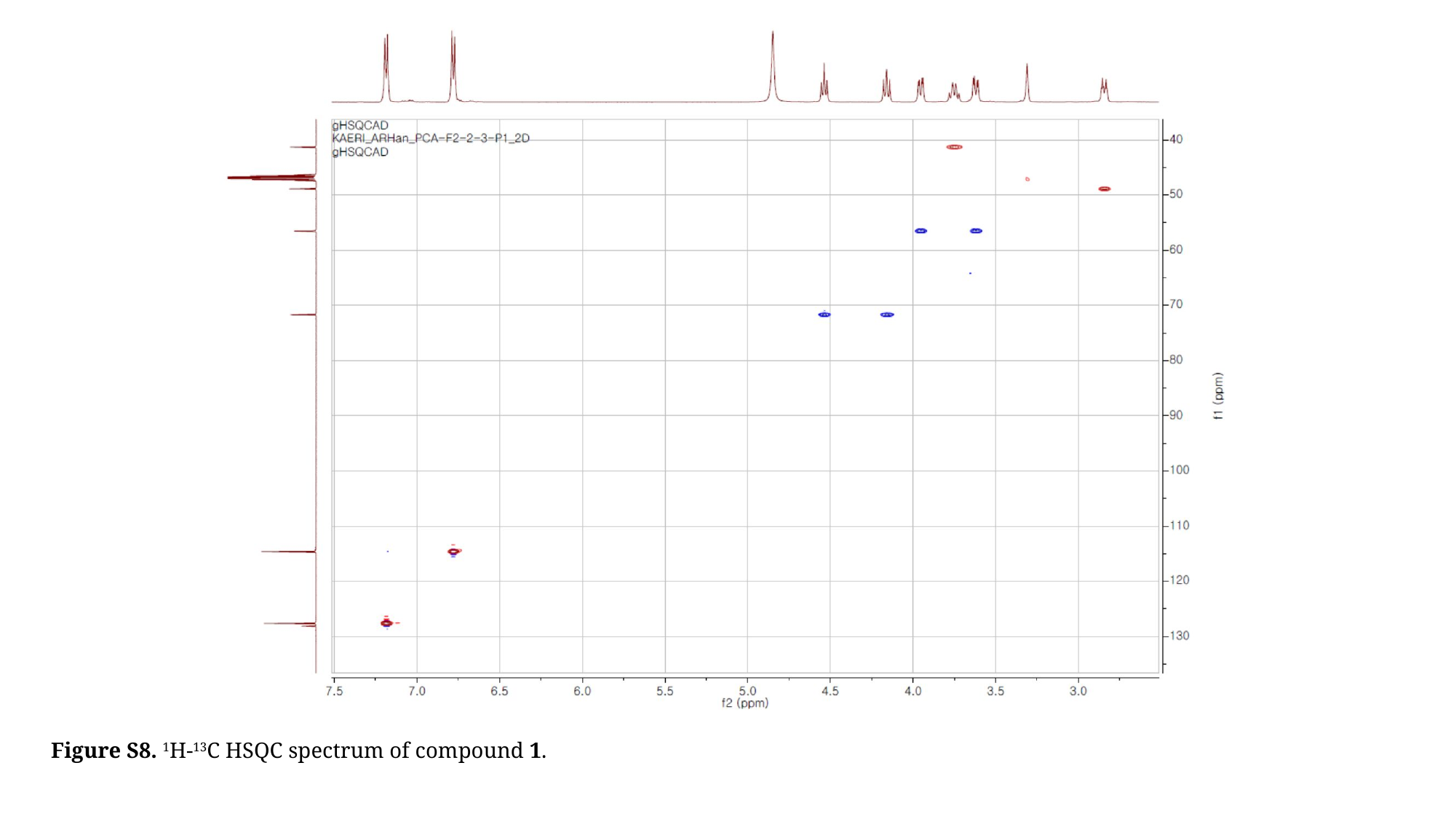

Figure S8. 1H-13C HSQC spectrum of compound 1.

## Slide 11
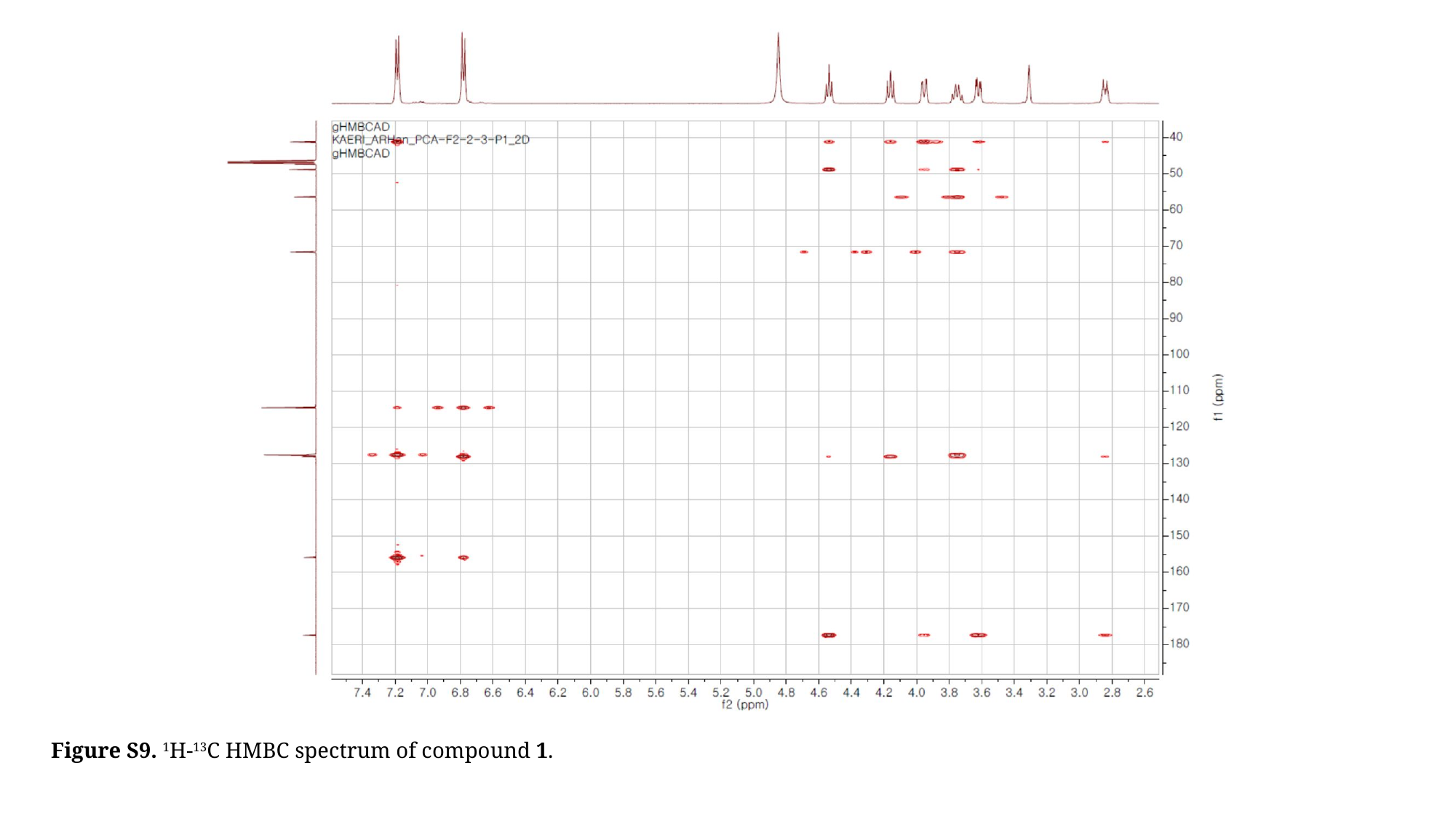

Figure S9. 1H-13C HMBC spectrum of compound 1.

## Slide 12
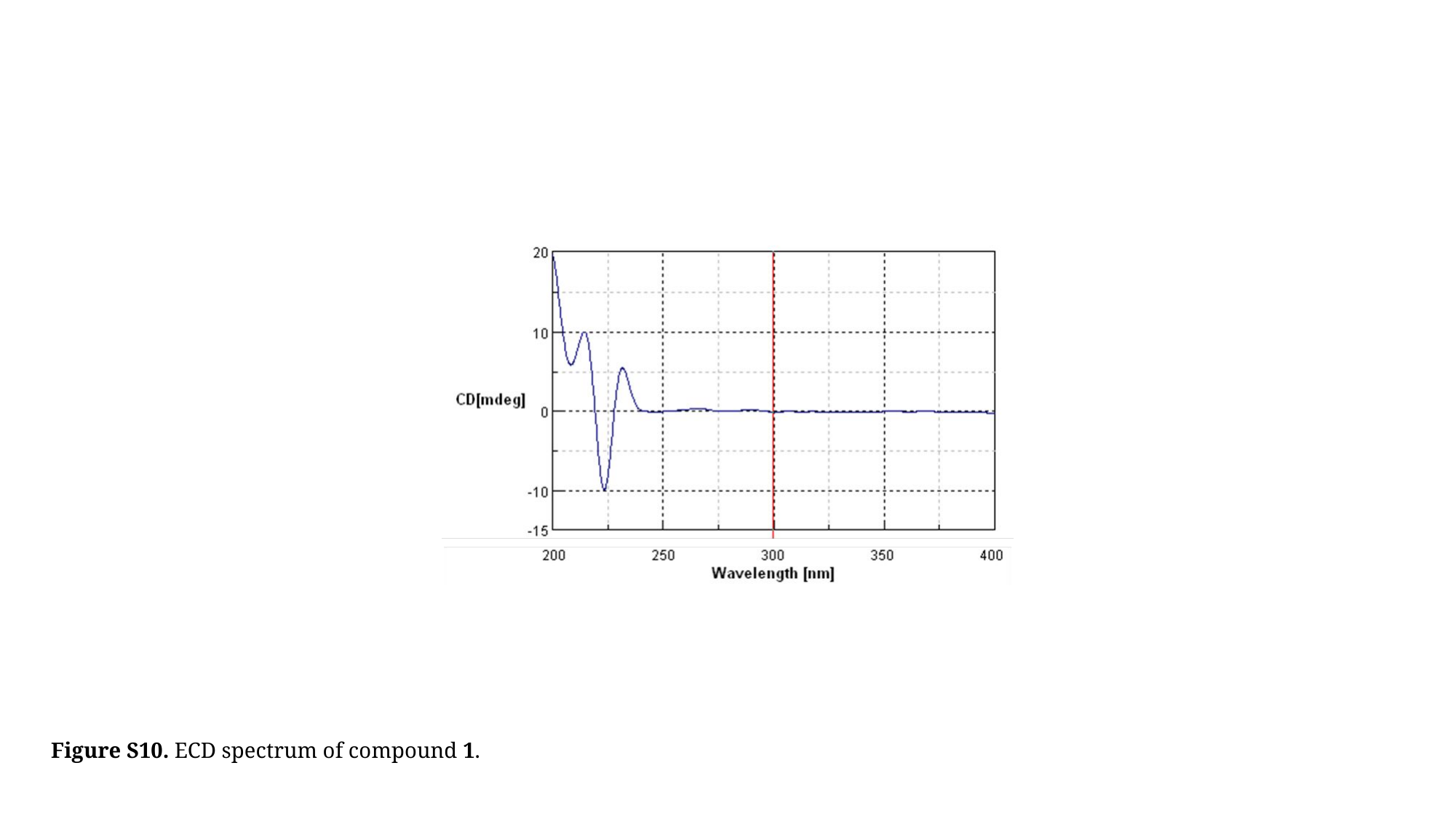

Figure S10. ECD spectrum of compound 1.

## Slide 13
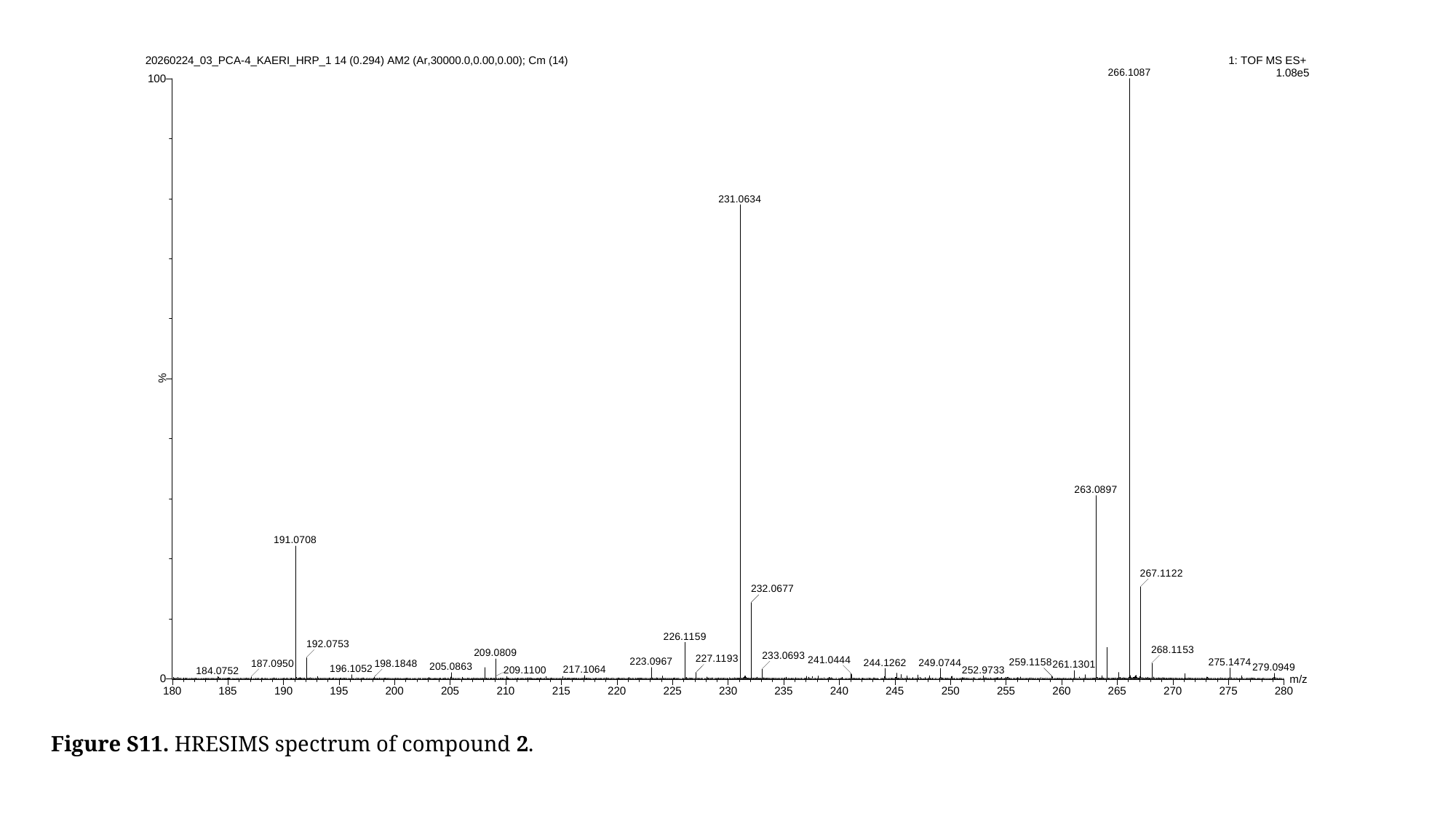

Figure S11. HRESIMS spectrum of compound 2.

## Slide 14
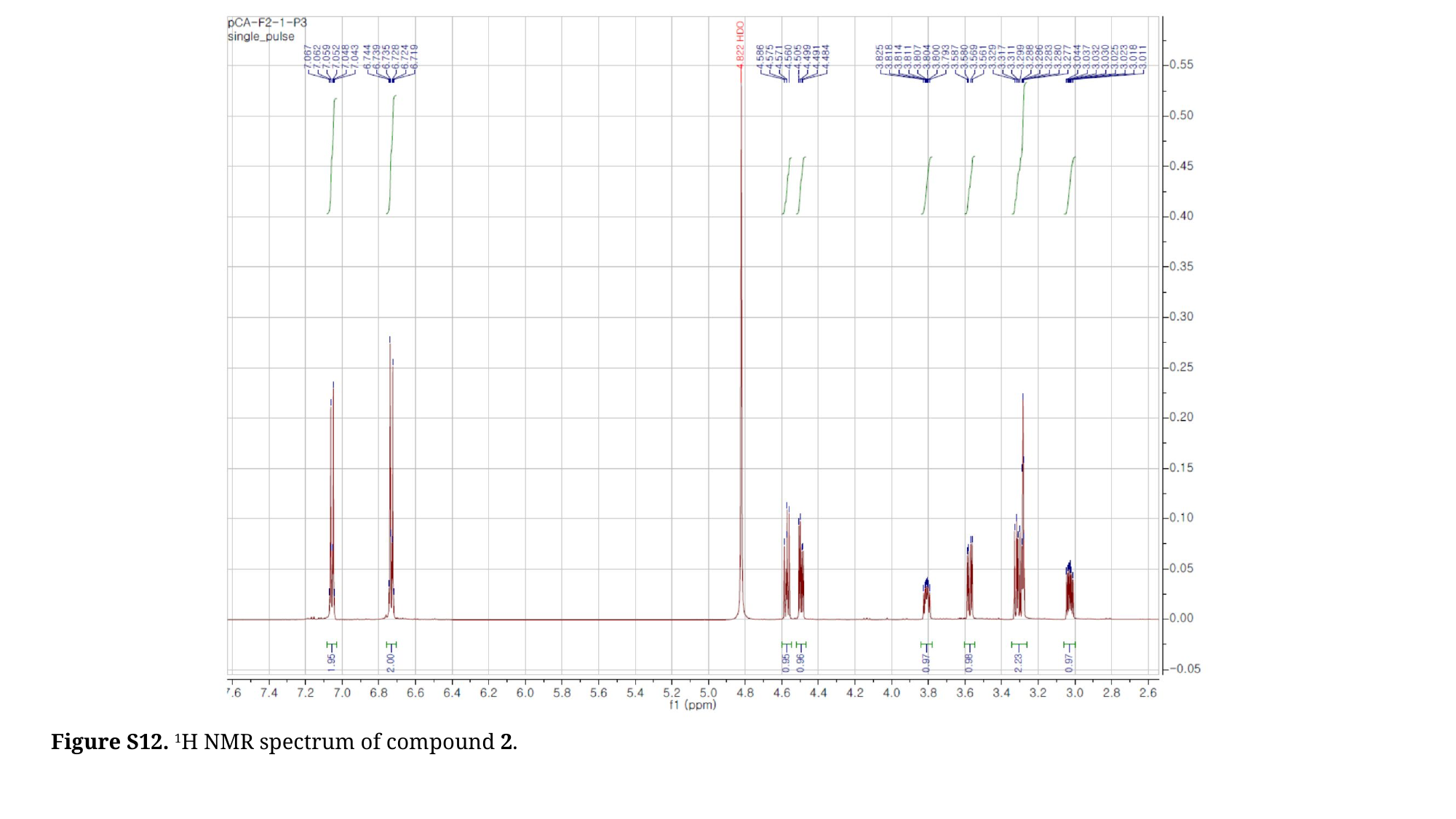

Figure S12. 1H NMR spectrum of compound 2.

## Slide 15
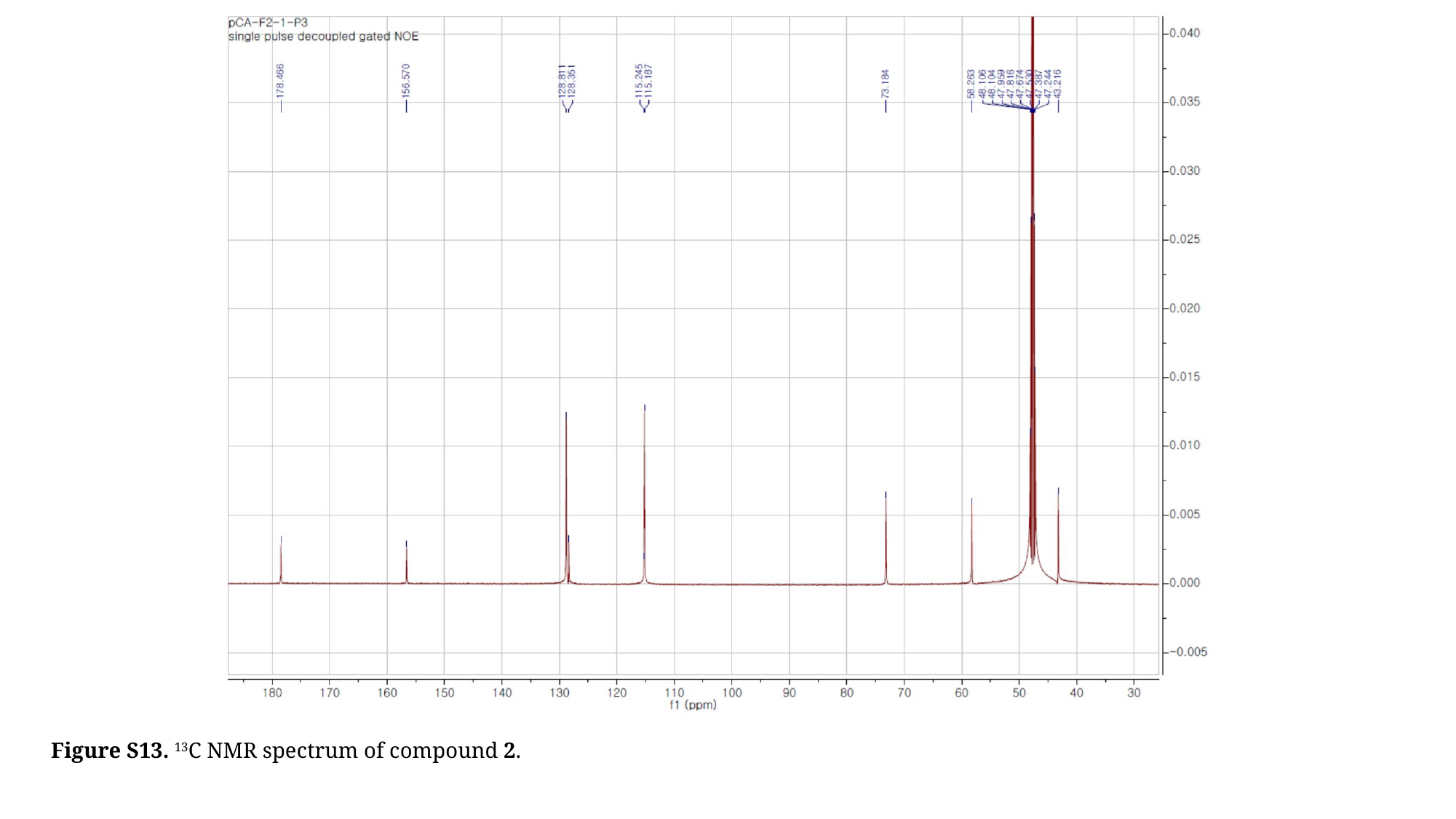

Figure S13. 13C NMR spectrum of compound 2.

## Slide 16
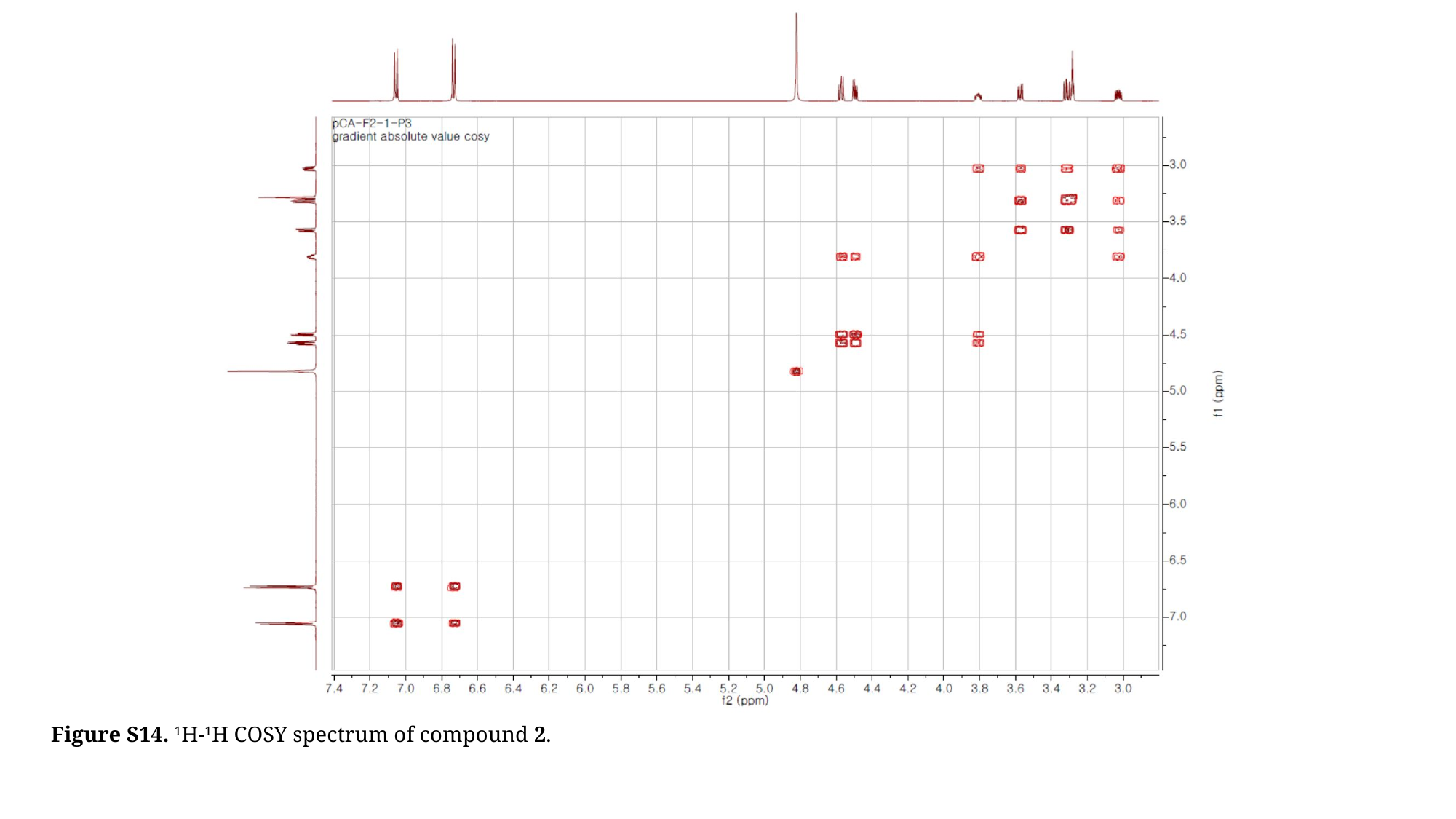

Figure S14. 1H-1H COSY spectrum of compound 2.

## Slide 17
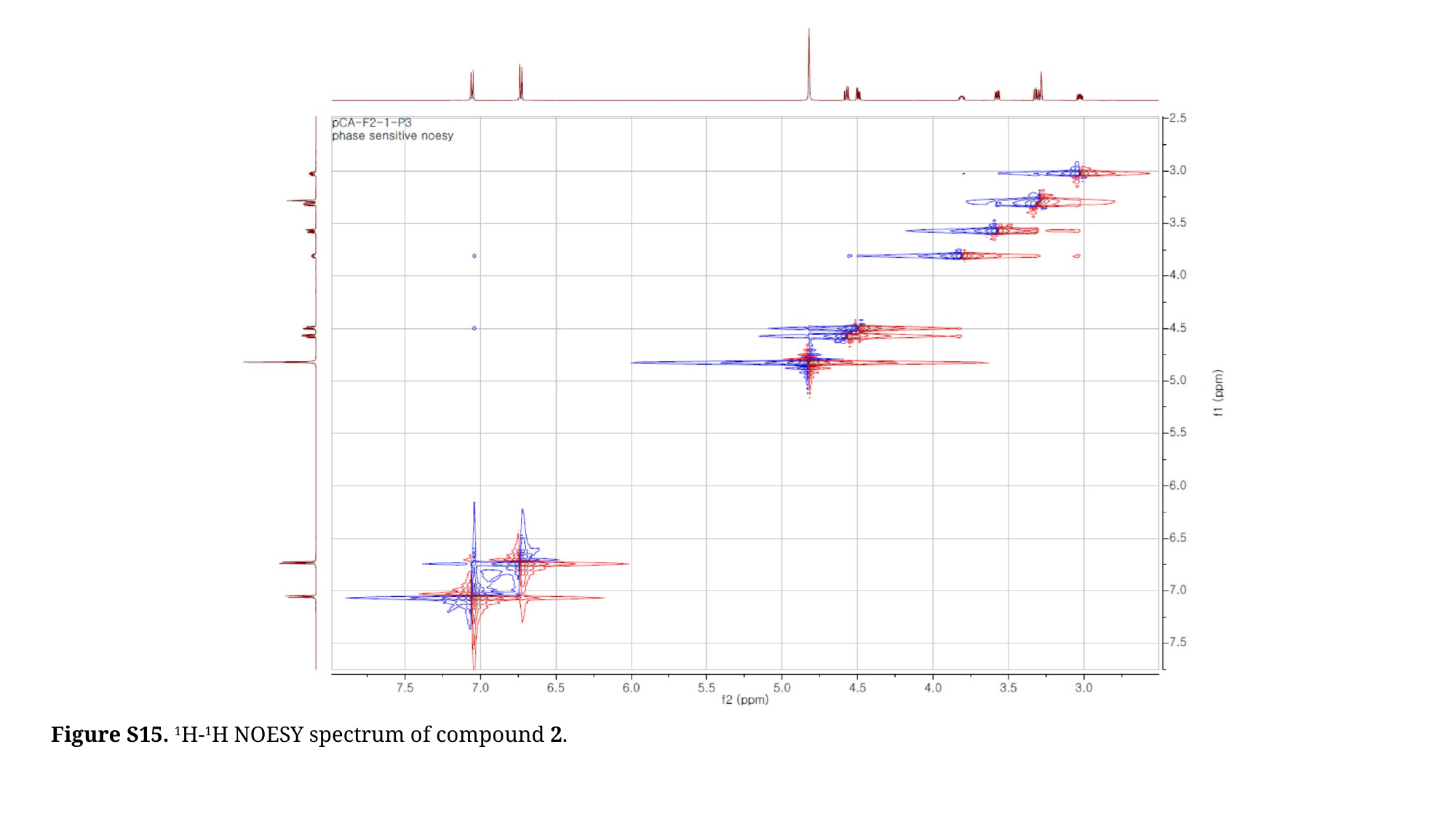

Figure S15. 1H-1H NOESY spectrum of compound 2.

## Slide 18
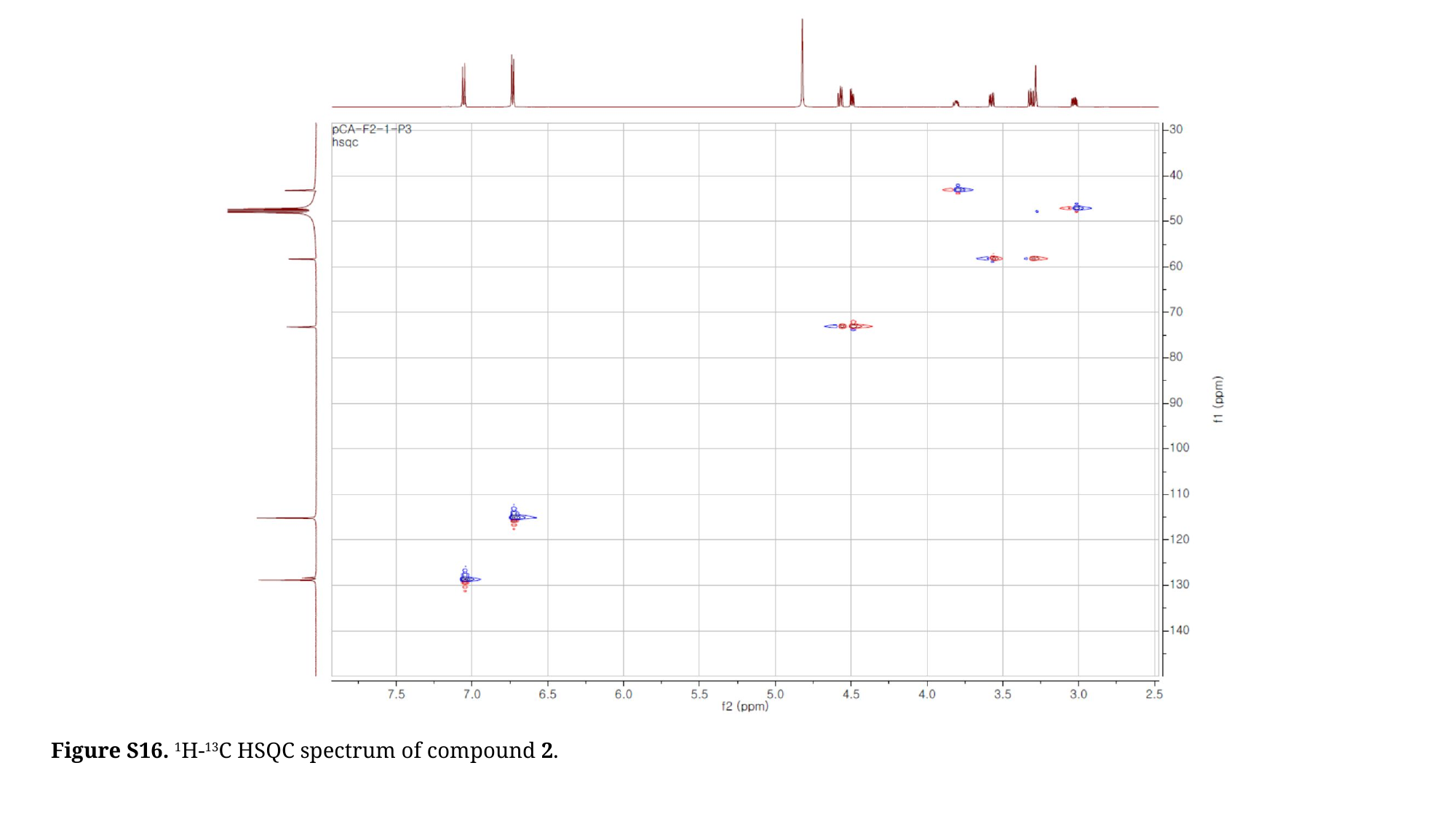

Figure S16. 1H-13C HSQC spectrum of compound 2.

## Slide 19
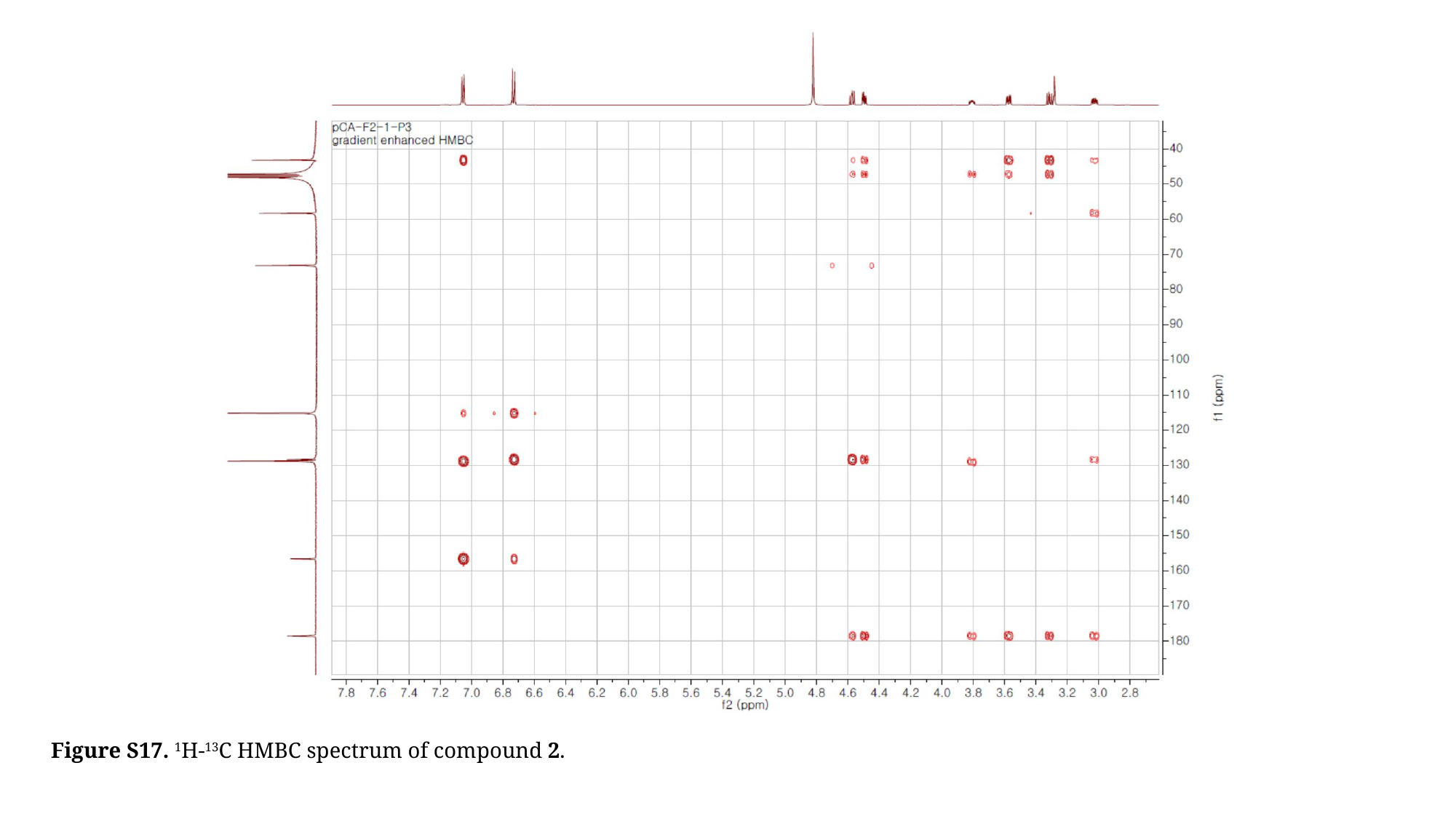

Figure S17. 1H-13C HMBC spectrum of compound 2.

## Slide 20
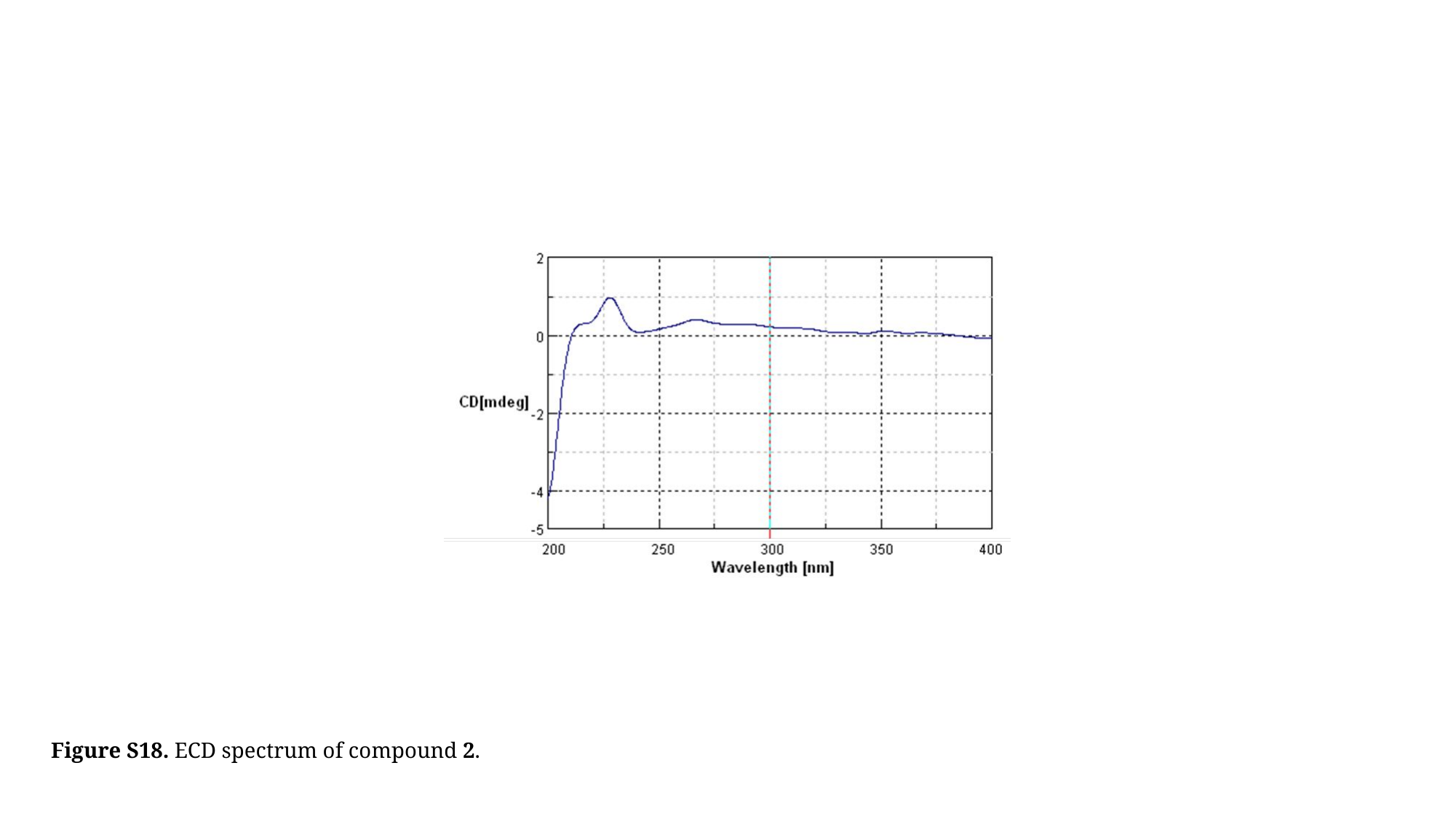

Figure S18. ECD spectrum of compound 2.

## Slide 21
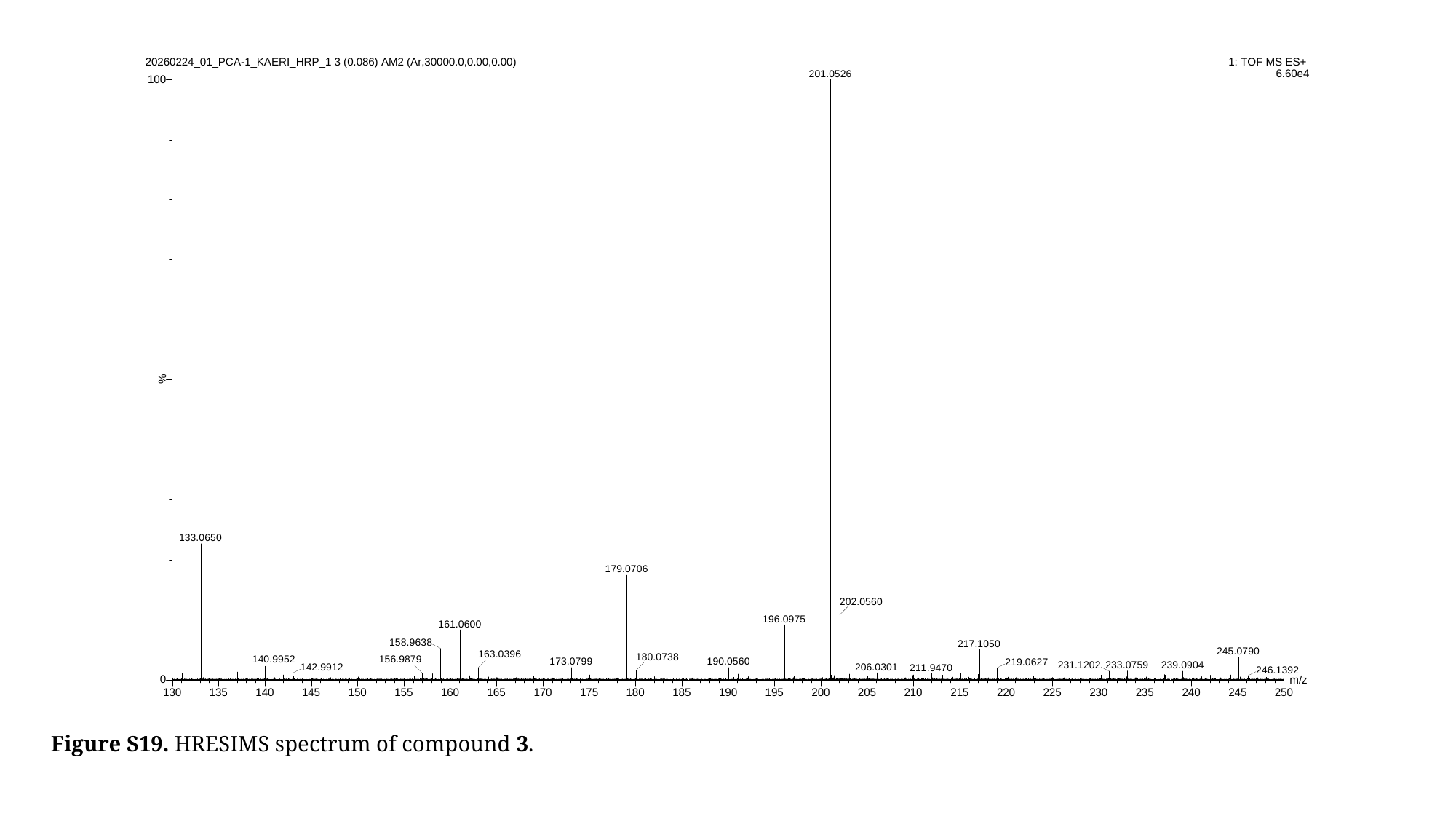

Figure S19. HRESIMS spectrum of compound 3.

## Slide 22
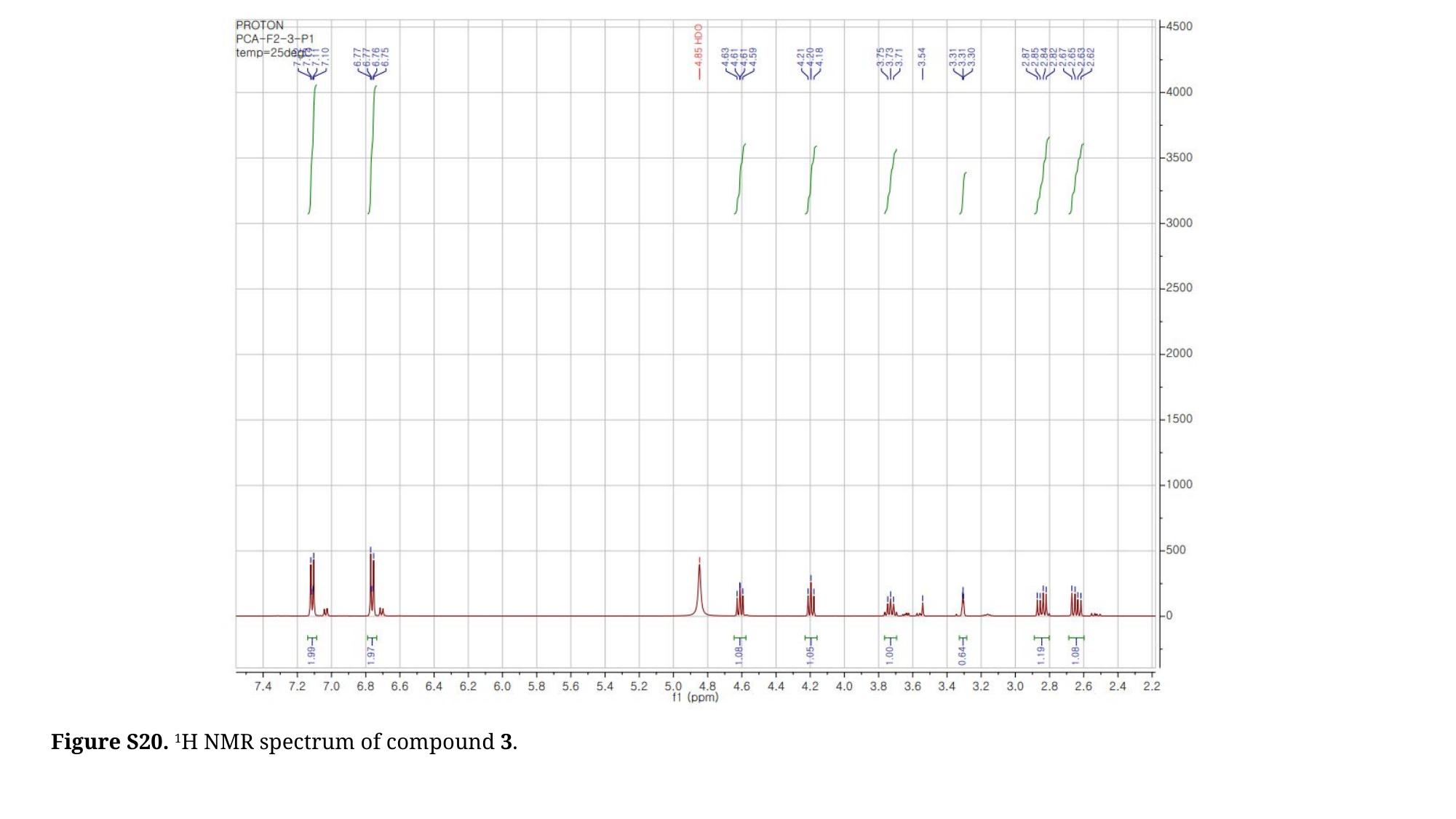

Figure S20. 1H NMR spectrum of compound 3.

## Slide 23
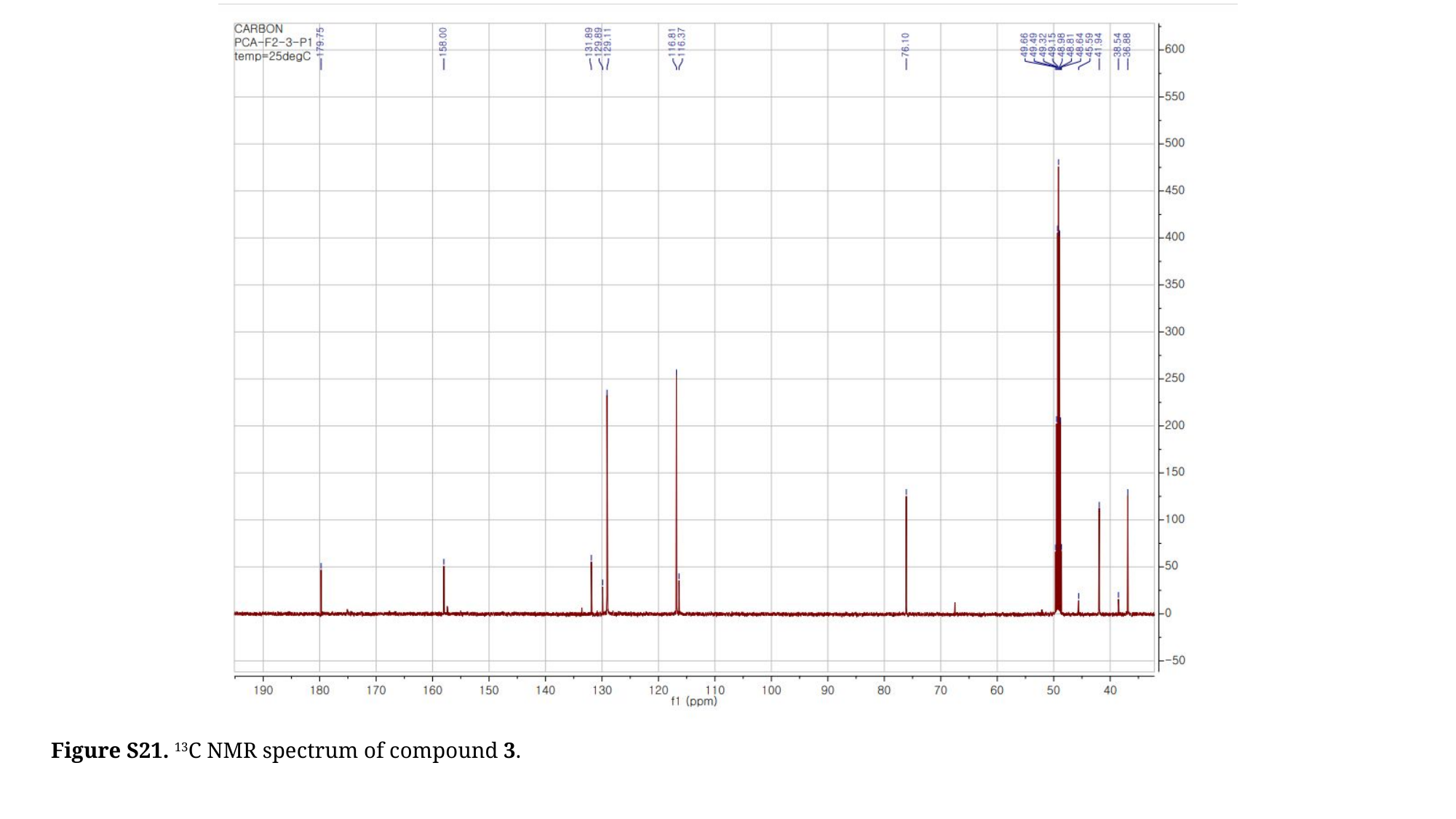

Figure S21. 13C NMR spectrum of compound 3.

## Slide 24
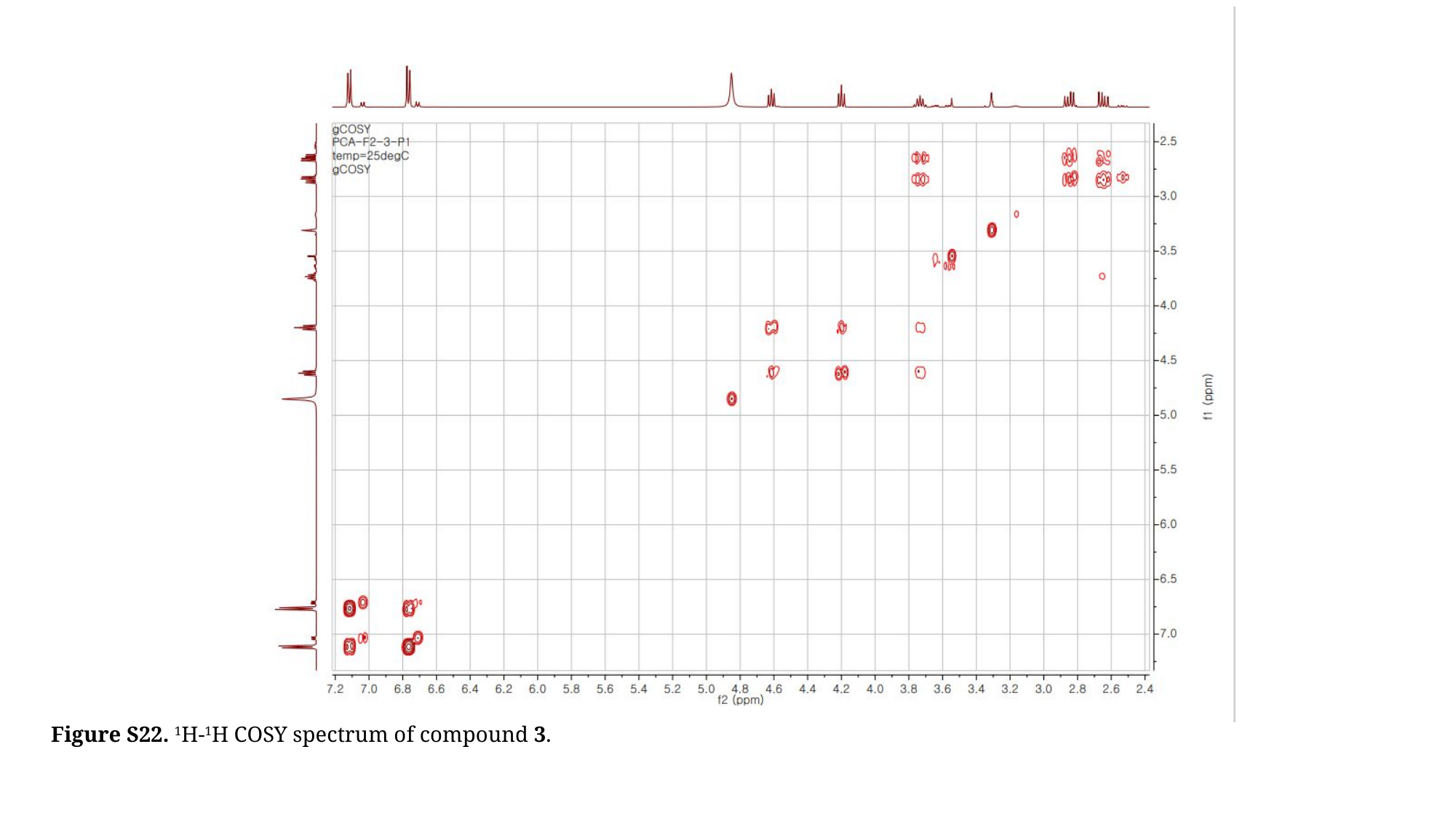

Figure S22. 1H-1H COSY spectrum of compound 3.

## Slide 25
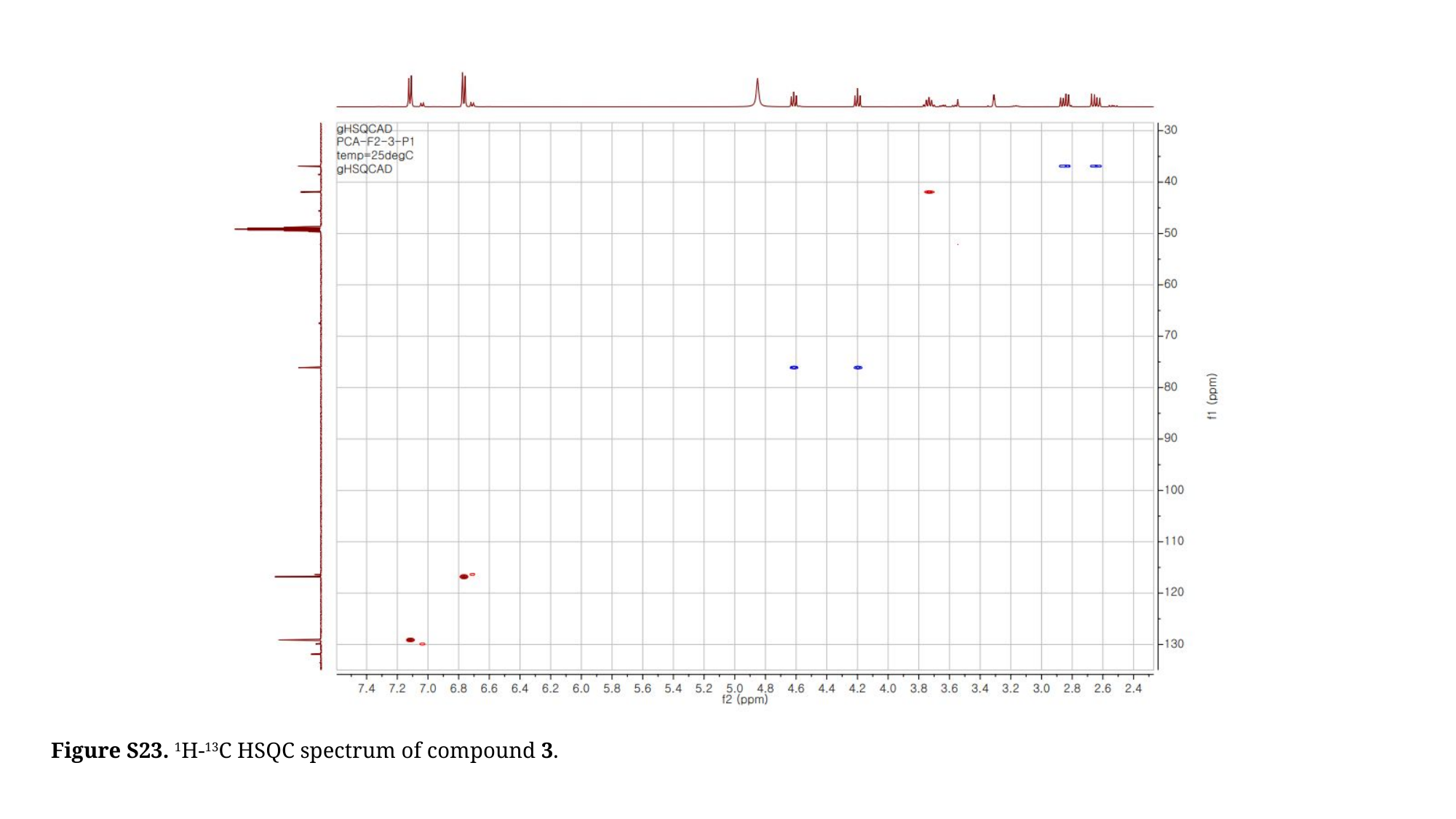

Figure S23. 1H-13C HSQC spectrum of compound 3.

## Slide 26
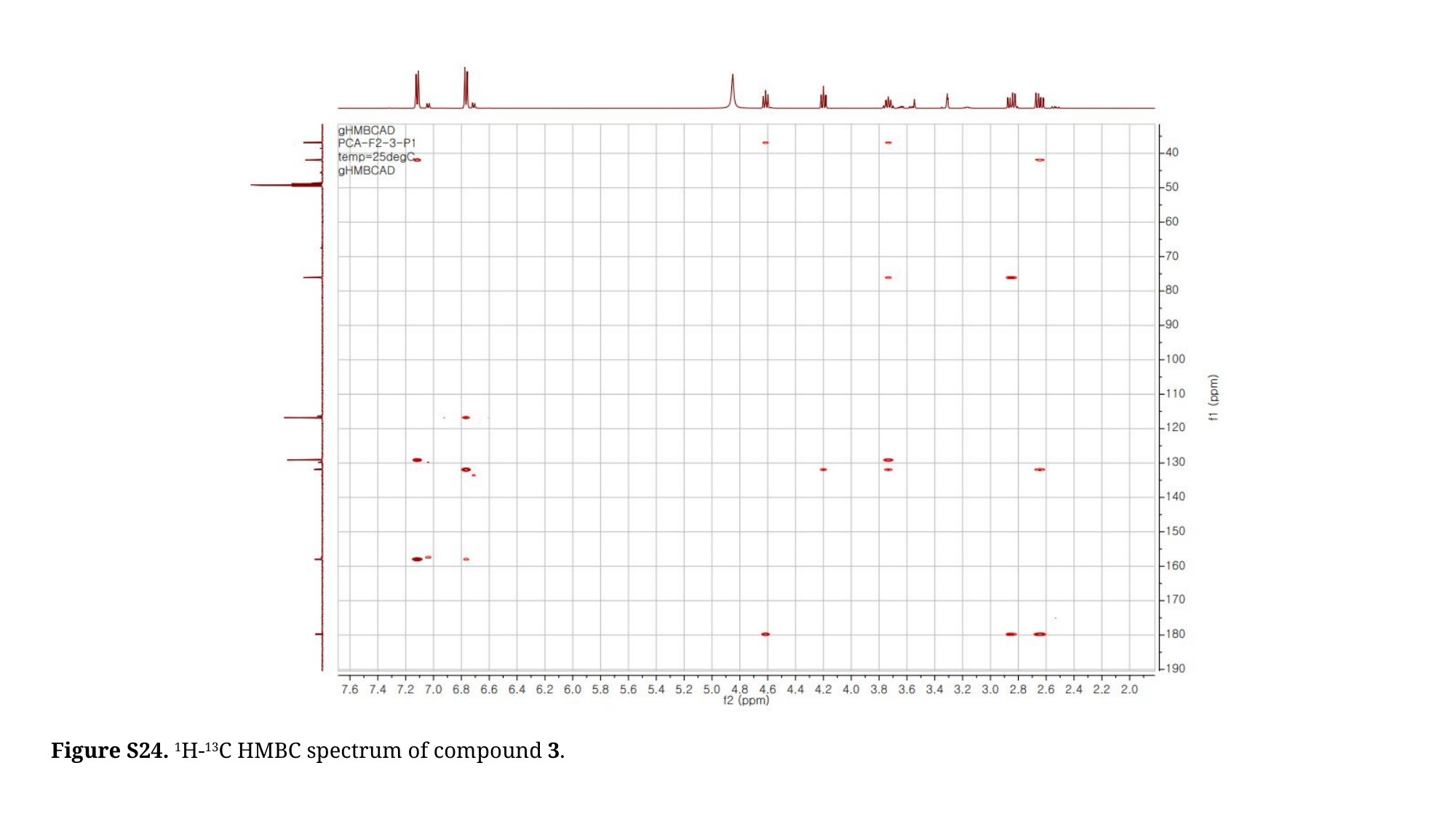

Figure S24. 1H-13C HMBC spectrum of compound 3.
